# At the intersection of soundscapes and roads: Quantifying anthrophony's influence on wildlife crossing structure use

**DOI:** 10.1002/eap.70192

**Published:** 2026-02-19

**Authors:** Thomas J. Yamashita, Ashley M. Tanner, Evan P. Tanner, Daniel G. Scognamillo, Michael E. Tewes, John H. Young, Jason V. Lombardi

**Affiliations:** ^1^ Caesar Kleberg Wildlife Research Institute, Texas A&M University – Kingsville Kingsville Texas USA; ^2^ Environmental Affairs Division, Texas Department of Transportation Austin Texas USA; ^3^ Wildlife Health Laboratory, California Department of Fish and Wildlife Rancho Cordova California USA; ^4^ Present address: Department of Fish, Wildlife, and Conservation Biology Colorado State University Fort Collins Colorado USA; ^5^ Present address: Rocky Mountain Research Station, United States Forest Service Fort Collins Colorado USA; ^6^ Present address: Safari Club International Foundation San Antonio Texas USA

**Keywords:** anthrophony, normalized difference soundscape index, road ecology, sound pressure level, Virginia opossum, wildlife crossing structures

## Abstract

Anthropogenic noise (anthrophony) can have significant negative effects on wildlife, causing both physiological (i.e., increased stress hormone production) and behavioral (i.e., altered anti‐predator behaviors, space use, or diel activity) changes in individuals. Roads are a major source of anthrophony, often contributing the most to the anthrophony in rural areas. Most efforts to reduce road effects on wildlife have focused on decreasing road‐associated mortality through the construction of wildlife crossing structures (WCSs) with little consideration for the anthrophony associated with these structures. Given the impacts of anthrophony on wildlife behavior, the effectiveness of WCSs could be altered without consideration of noise pollution. Therefore, understanding how anthrophony is structured in space and time and how it impacts WCS use is an important aspect of assessing the effectiveness of WCSs. We developed a framework for assessing anthrophony at WCS using an array of autonomous recording units to monitor overall acoustic conditions. We then examined how wildlife crossing rates were associated with anthrophony using camera traps. We monitored five underpass‐style WCSs built in the Lower Rio Grande Valley of South Texas, USA, using camera traps and acoustic recording units. We measured sound pressure level (SPL [dB]) and relative level of anthrophony (using the normalized difference soundscape index [NDSI]) at six positions around each WCS: two at elevation (road grade) with the road surface (west and east), two at the WCS entrances, and two in the middle of the WCSs. We then used SPL and NDSI to predict the probability of a successful crossing by Virginia opossum (*Didelphis virginiana*), a common, disturbance‐tolerant mammal. While the relative amount of anthrophony did not differ, smaller WCSs and those with less traffic were up to 40 dB quieter than larger WCSs and those with more traffic. Opossums spent more time at WCSs when it was quieter on average and were more likely to successfully cross through a WCS when there was less vehicle noise. Our study highlights the importance of considering soundscapes in assessing WCS effectiveness and represents a framework that can be used for further exploration of the impacts of anthrophony on WCS use.

## INTRODUCTION

Noise in the environment has drastically increased since the industrial revolution due to human population growth, urbanization, and the expansion of transportation networks (Shannon et al., 2016). Anthropogenic sounds (or anthrophony) have become a significant component of natural soundscapes (the acoustic profile of a landscape), which were previously composed exclusively of biological sounds (biophony; such as bird song, frog noises, and mammal calls) and geophysical‐derived sounds or geophony such as wind, rivers, and rain (Pijanowski, Farina, et al., 2011; Pijanowski, Villanueva‐Rivera, et al., 2011). Anthrophony, especially from vehicle traffic and energy development (Pijanowski, Villanueva‐Rivera, et al., 2011), is often described as undesired sound (or noise) in natural soundscapes (Villanueva‐Rivera et al., 2011). Anthrophony has been shown to have numerous negative impacts on animals (Blickley & Patricelli, 2010). In noisy environments, animals may experience greater levels of stress hormones, have greater susceptibility for disease, avoid particularly noisy areas either completely or for parts of the day, experience difficulties communicating with conspecifics, or move more quickly through noisy compared to quieter areas, all of which likely reduce fitness (Berkhout et al., 2023; da Silva et al., 2023; Francis & Barber, 2013; Kok et al., 2023; McClure, 2021; Shannon et al., 2016).

Roads are a major source of anthrophony; yet, most studies of road impacts on wildlife focus on the direct threat of vehicle collisions, habitat loss, or the fragmentation effect caused by high traffic (Andrews et al., 2015; Forman et al., 2003; van der Ree et al., 2015). Vehicle noise typically occurs at relatively low frequencies (<2 kHz) and produces 75–95 dB of sound at 15–20 m (Table 1; Schomer, 2000, Şabikoğlu & Akbaba Şabikoğlu, 2021) which likely has a strong impact on animal behavior around roads (Collins et al., 2022) through sound masking or increased antipredator behaviors (Francis & Barber, 2013). On high‐traffic roads, vehicle traffic creates conditions of chronic noise, while on lower traffic roads, traffic tends to occur in bursts (acute noise), creating intermittent noise pollution that may impact otherwise quiet areas (Shilling et al., 2020). Road mitigation structures for wildlife are frequently used to reduce road mortality and increase landscape connectivity (Helldin, 2022). However, these structures are expensive (Huijser et al., 2022), and are often placed in areas where they are thought to be most effective, including along migration routes (Gagnon et al., 2011) and near areas of high numbers of vehicle collisions (Grilo et al., 2015; Teixeira et al., 2017).

**TABLE 1:** Sound pressure level of different sources of anthropogenic noise in decibels at 15–20 m from the source (Şabikoğlu & Akbaba Şabikoğlu, 2021; Schomer, 2000).

Anthropogenic noise	Sound pressure level (dB)
Threshold of human hearing	0
Rustling leaves	20
Wind turbines, normal conversation	50–60
Standard automobile	75
Commercial truck	90
Jet engine	150

Typical wildlife crossing structures (WCSs) aim to provide safe passage for wildlife across roads and do not address the effects of noise pollution on WCS efficacy (Clevenger, 2005). While noise has been shown to be negatively correlated with crossing rates (Clevenger & Waltho, 2005), anthrophony is rarely considered in WCS design. Consideration of road noise impacts would provide important insights into the effectiveness of WCSs and can inform future WCS construction. Because anthrophony is rarely considered in their design, WCSs may create acoustic conditions that amplify vehicle noise and deter animals. For example, large, open, bridge‐style WCSs, while known to be important and effective for many large‐bodied species, may allow more sound into the WCS (Shilling et al., 2022), which may scare some animals from the structures compared to smaller box culverts, which have more barriers to sound. Additionally, high variability in vehicle traffic makes noise at WCSs unpredictable, and reduced visual cues when around a WCS may make noise, particularly acute noise, especially disturbing to wildlife (Francis & Barber, 2013). Additionally, vehicle noise likely impacts WCS use at finer spatial and temporal scales than most studies have previously examined.

Physical barriers, temperature, relative humidity, and wind speed are factors that can impact how sound travels through the environment (Hardy et al., 1942). Physical barriers, including human structures, topography, and vegetation, can actively block and diffuse sound, reducing the amplitude of sounds that reach a particular site (Evans & Cooper, 2012). These factors might mitigate noise impacts at WCSs, promoting use in and around WCSs (Shilling et al., 2022). Temperature and relative humidity can affect the density of air, which impacts the speed of sound in air and, therefore, could impact the amplitude (volume) of a particular signal at a given location (Bohn, 1988; Harris, 1966). Sound travels faster in less dense air, so when it is warmer and more humid, noises are often louder (Prospathopoulos & Voutsinas, 2005) and therefore likely to have a greater impact on wildlife. Microclimate conditions at WCSs are likely to be important in determining how noise travels through WCSs and therefore how noise might influence WCS use. Wind can have varying effects on noise propagation, depending on the direction and speed of the wind. When wind speed is higher, wind noise may mask vehicle noise because both sounds occur at similar frequencies (typically 1–2 kHz; Prospathopoulos & Voutsinas, 2005). Wind can also carry sounds, potentially amplifying road noise at a WCS (Evans & Cooper, 2012).

Sound can be measured in a variety of ways, but acoustic indices derived from long‐duration recordings of the environment provide an ideal platform for measuring and quantifying the soundscape around a WCS (Towsey, 2018). Acoustic indices summarize parts of the acoustic spectrogram of a recording to numerically quantify characteristics of sound in the environment (Towsey et al., 2014). Many acoustic indices capture characteristics of sound in the environment, including anthrophony (Alcocer et al., 2022), such as sound pressure level (SPL) and the normalized difference soundscape index (NDSI; Fuller et al., 2015). These two indices provide different metrics of the soundscape; yet, both are informative in assessing how anthrophony impacts WCS use. SPL measures the force of a signal in decibels and is often calculated in reference to the minimum level of human hearing (20 μPa; Table 1). This makes it an ideal measurement for determining how loud a location is, an important consideration in determining WCS effectiveness (Collins et al., 2022). Anthrophony typically occurs in the 1–2 kHz frequency range while biophony typically occurs at higher frequencies (2–20 kHz). SPL, when measured only at lower frequencies, can effectively capture road noise because vehicles are often the loudest sources of noise on the landscape. The NDSI represents the proportion of the total sound that is biological in origin compared to the sound that is anthropogenic in origin, making it an ideal proxy for the amount of anthrophony in a recording (Fairbrass et al., 2017; Kasten et al., 2012).

We aimed to develop a framework for assessing if and how anthrophony may alter WCS effectiveness to inform future WCS design. Using a common, disturbance‐tolerant mammal (Virginia opossum; hereafter opossum; *Didelphis virginiana*) to test this framework, we aimed to understand how WCS structure impacted noise propagation and how vehicle noise impacts WCS use. We had two main objectives for this study: (1) assess the acoustic profile of WCSs to identify differences in noise levels within WCSs and (2) assess how animal crossing success and time spent at WCSs were affected by spatiotemporal variability in anthrophony and microclimate conditions. We hypothesized that (1) anthrophony would be lower in the center of a WCS compared to the outside, (2) larger and more open WCSs would be louder than smaller ones, and (3) the side of the WCSs most exposed to prevailing winds (east in our specific study) would have lower levels of anthrophony as a result of wind masking and lack of wind‐driven noise propagation. Additionally, we expected that crossing success would increase when there was less anthrophony. Finally, we predicted that the duration that an animal spends at a WCS would increase when SPL decreased and NDSI increased (representing a decrease in the total sound and anthrophony at a given time).

## METHODS

### Study area

**FIGURE 1:**
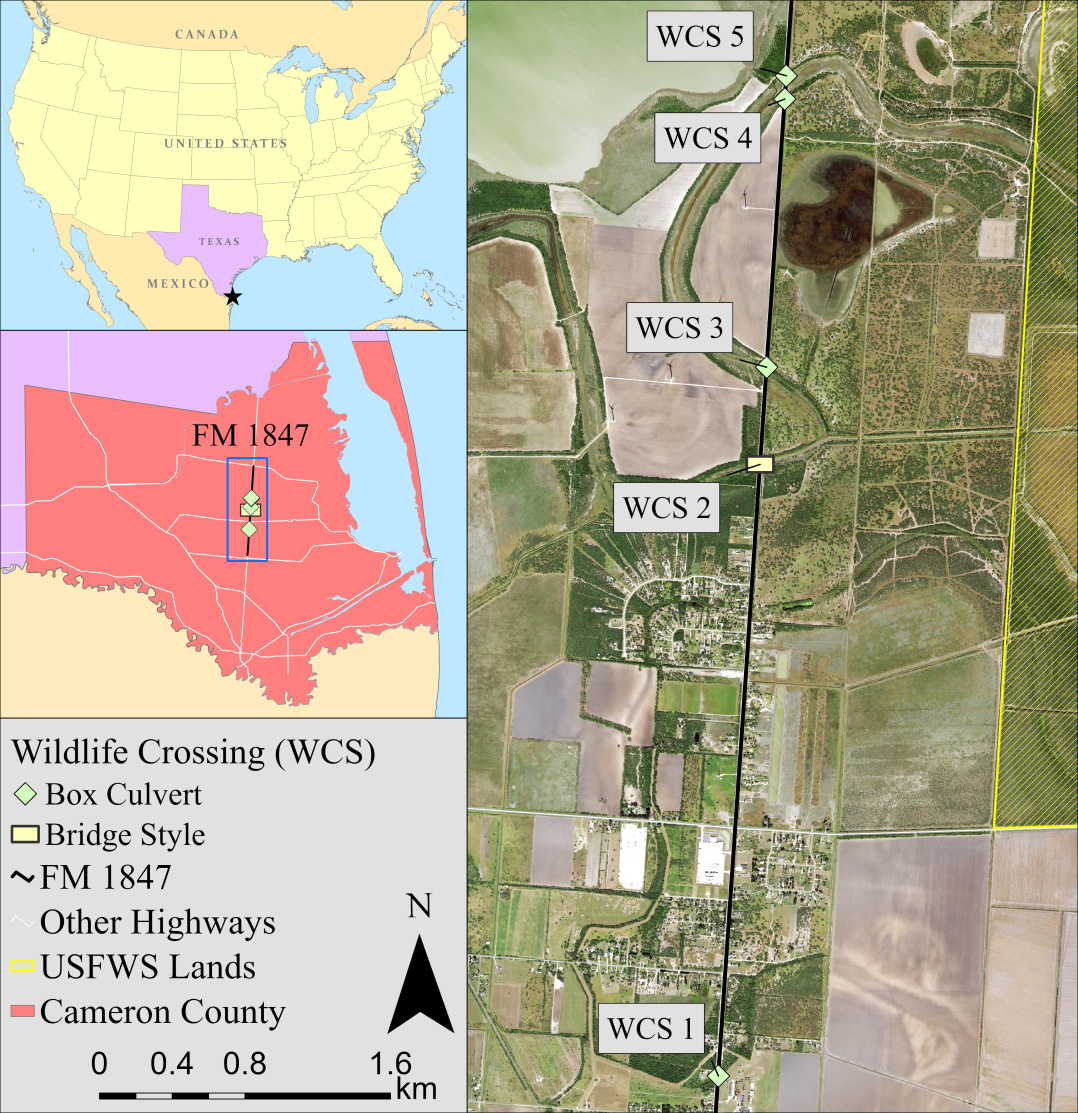
Study area showing the location of the five wildlife crossing structures (WCSs) where acoustic recording devices were set up to monitor anthropogenic noise in fall 2023 and spring 2024 along Farm‐to‐Market (FM) 1847 in Cameron County, Texas, USA. The basemap is freely available from the national agriculture imagery program through the US Department of Agriculture.

**FIGURE 2:**
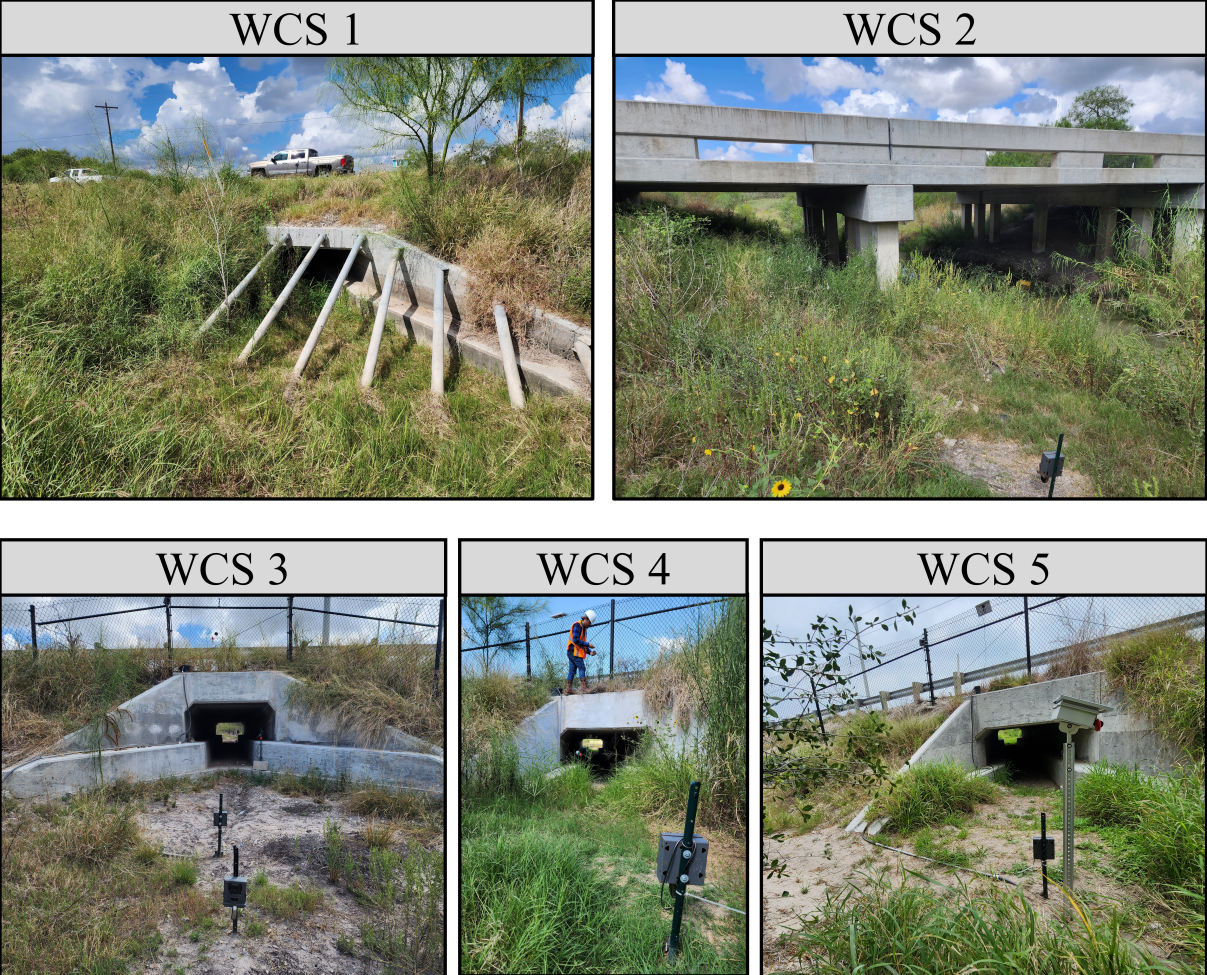
Photos of the five wildlife crossing structures (WCSs) constructed on Farm‐to‐Market (FM) 1847 in Cameron County, Texas, USA. Photo credits: T. J. Yamashita.

Our study took place in the Lower Rio Grande Valley, Texas, USA (Figure 1), a region that is experiencing increasing human population growth, resulting in high levels of urban expansion and road construction (Lombardi et al., 2020). The region is part of the North American Coastal Plain biodiversity hotspot (Noss et al., 2015) and is home to many endangered and threatened species, including the ocelot (*Leopardus pardalis*; Martinez et al., 2024; U.S. Fish & Wildlife Service, 2016). Roads are a major source of mortality for ocelots, so road mitigation structures, including WCSs, are being built throughout southern Texas for ocelot conservation (Blackburn et al., 2022). One road where WCSs have been built is Farm‐to‐Market (FM) 1847 in Cameron County, Texas, located within the geographic range of this ocelot population (Scognamillo et al., 2023). Five underpass‐style WCSs were built on FM 1847 between 2020 and 2022: four box culverts and one bridge‐style WCS (Figure 2, Table 2). Each WCS has different dimensions, urbanization levels, and land use/landcover characteristics, which all impact how wildlife use the WCSs (Yamashita, Scognamillo, et al., 2025). The study area was dominated by low‐intensity urban, row‐crop agriculture, ranchland, and federally protected areas (e.g., Laguna Atascosa National Wildlife Refuge). The vegetation community includes Gulf cordgrass (*Spartina spartinae*) prairie, honey mesquite (*Neltuma glandulosa*) woodland, and Tamaulipan thornscrub communities (Elliott et al., 2014; Ewing & Best, 2004). Tamaulipan thornscrub is a diverse community of small to medium (<5 m height) thorny trees and shrubs, endemic to southern Texas and northeastern Mexico (Luera & Gabler, 2022; Mohsin et al., 2021). The region experiences hot and humid summers (average July temperature of 36°C) and mild winters (average January temperature of 10°C; Palecki et al., 2020) with periodic rainfall (on average 313–529 mm per year; Cooper & Wagner, 2013). Consistent tropical winds from the Gulf of Mexico create ideal conditions for wind energy production (Chang & Starcher, 2019), and the part of the study site is located within one such wind farm (Rand et al., 2020).

**TABLE 2:** Characteristics of wildlife crossing structures (WCSs) along Farm‐to‐Market road 1847 in Cameron County, Texas, USA showing the type of WCS (box culvert or bridge), dimensions in meters (openness ratio in parentheses is calculated as height [*H*] × width [*W*]/length [*L*]), vehicle traffic as measured in annual average daily traffic, the presence of water, and the density of buildings to represent urbanization in the area.

WCS	Crossing type	Dimensions (H × W × L)	Vehicle traffic^a^	Water presence	Building area^b^
WCS1	Concrete box culvert with a concrete step	2.13 × 1.22 × 24.38 (0.107)	2753	No	0.0153
WCS2	Bridge‐style WCS with a dirt bench	14.63 × 5.35 × 24.38 (3.211)	2050	Permanent	0.0039
WCS3	Concrete box culvert with a concrete step	2.13 × 1.52 × 18.29 (0.178)	2050	No	0.0005
WCS4	Concrete box culvert with a concrete step	2.13 × 1.52 × 17.98 (0.181)	2050	No	0
WCS5	Concrete box culvert with a concrete step	2.13 × 1.52 × 21.03 (0.155)	2050	No	0

^a^ Annual average daily traffic (average number of vehicles per day over the course of 1 year) is from Yamashita, Scognamillo, et al. (2025) and Texas Department of Transportation (2022).

^b^ Building area for these WCSs is calculated as the proportion of area occupied by buildings within 1 km of the WCS and is available in Yamashita et al. (2023) and Yamashita, Scognamillo, et al. (2025).

### Data collection

#### Acoustic profile of WCSs

**FIGURE 3:**
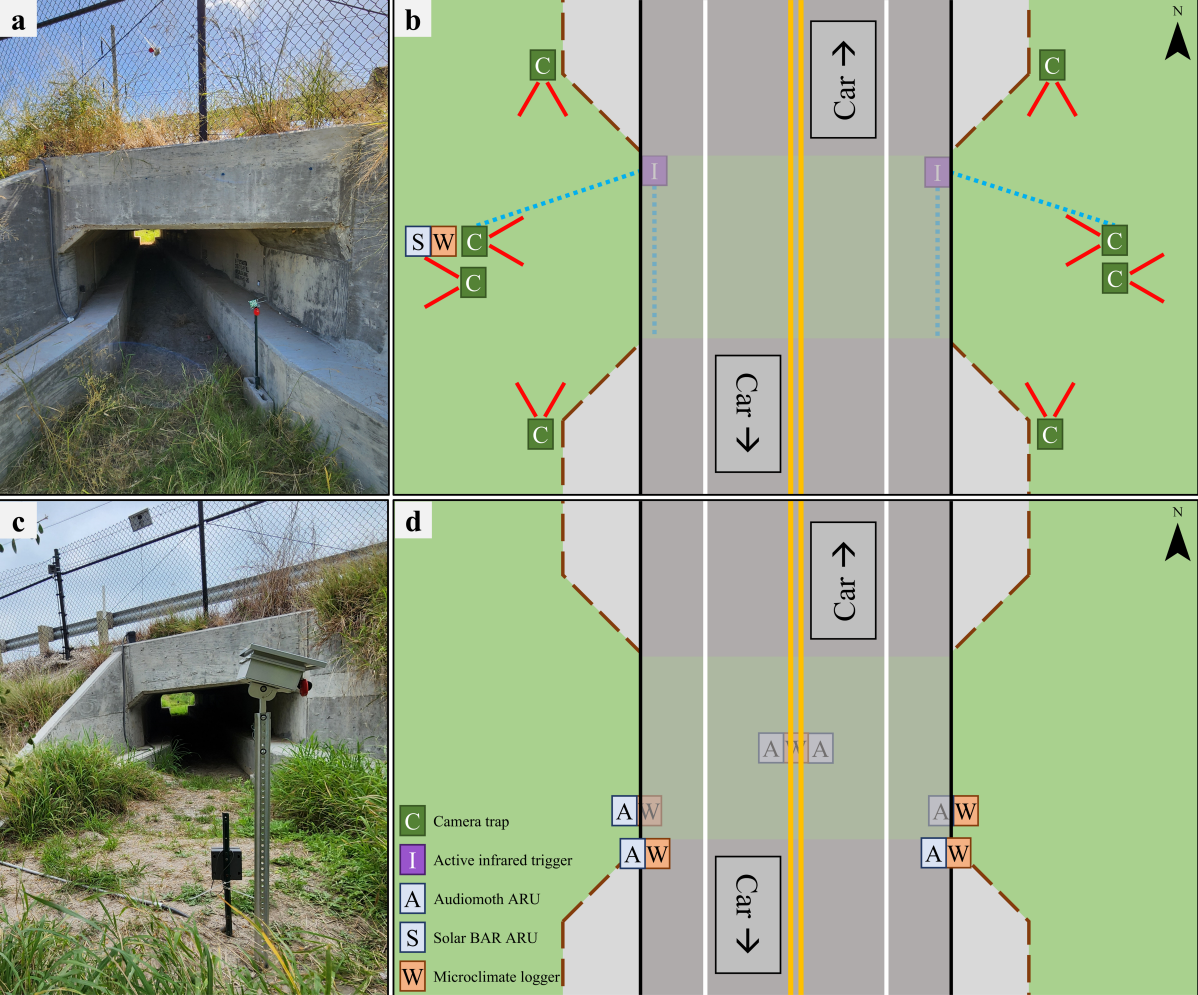
Placement of acoustic recording units (ARUs) along Farm‐to‐Market (FM) 1847 in fall 2023 and spring 2024 in Cameron County, Texas, USA used to (a, b) determine the acoustic profile around a wildlife crossing structure (WCS) and (c, d) assess how anthropogenic noise impacts Virginia opossum (*Didelphis virginiana*) use of WCSs. Red lines represent the direction and approximate field of view of the cameras. Dashed blue lines represent the active infrared trigger used to aid in capture of WCS use. Dashed brown lines represent the WCS. Photo credits: T. J. Yamashita.

We deployed six Audiomoth (Hill et al., 2018, 2019) acoustic recording units (ARUs) around each of the five WCSs (Figure 3a) to assess the acoustic profile of WCSs (hereafter position analysis). Audiomoths have directional microphones, so we placed ARUs towards the primary source of anthrophony (i.e., towards the road). One device was placed at grade on each side of FM 1847, facing the road, one device was placed at each entrance of the WCS facing the outside (away from the WCS), and two devices were placed in the center of the WCS, facing each entrance. We programmed ARUs to record at 8 kHz to isolate anthropogenic noises and record continuously, taking 24‐h recordings, and restarting each day. The ARUs were deployed for 50 days between August and October 2023. We checked ARUs every 2 weeks to exchange batteries and memory cards. Due to numerous device failures, functionality was highly variable, and devices recorded for at least 1 h a day for an average of 27.27 ± 7.19 days.

We deployed a Kestrel Drop D2 (Nielson‐Kellerman Co., Boothwyn, PA, USA) device at each WCS to record ambient temperature (in degree Celsius, accuracy ±0.4°C) and relative humidity (in percentage, accuracy ±1%) at each ARU location. The Kestrels were omnidirectional, so we only placed one device in the center of the WCS. Microclimate conditions were recorded every 10 min, and we retrieved the logs when we checked the ARUs.

#### Impact of anthrophony on WCS use

At the same WCSs, we used camera traps to monitor the WCSs for multiple concurrent studies (Lombardi et al., 2022; Scognamillo et al., 2023; Yamashita et al., 2024; Yamashita, Lombardi, et al., 2025; Yamashita, Scognamillo, et al., 2025). Eight to 12 Reconyx Hyperfire2 camera traps (Reconyx Inc., Holmen, WI, USA) were established at each WCS to capture use and describe animal response. Camera traps were placed 30–50 cm above the ground and programmed to take photos in three‐shot bursts with no delay between triggers. Additionally, an active infrared trigger was set up at WCS3, 4, and 5 to aid in the capture of crossing events. The active infrared trigger system utilizes an infrared beam set up at the entrance of each WCS to increase detection and identification accuracy (Cogan, 2018; Roy et al., 2024). We visited camera traps once a month to exchange memory cards and perform camera maintenance (change batteries, clear vegetation, etc.). Camera trap sites were also visited between memory card exchanges to clear vegetation that may interfere with camera functionality. For this study, we used camera trap data collected over 1 month from 16 March to 15 April 2024. This time period represented a period of high animal movement in South Texas (Roy et al., 2024) and therefore represented a likely time when animals would be using WCSs in the area.

One Frontier Labs Solar BAR (Frontier Labs Australia, Salisbury, QLD, Australia) ARU was set at each WCS to monitor anthrophony to assess how anthrophony impacts WCS use (hereafter, interactions analysis). We set the ARU on the west side of the road, near the WCS entrance (Figure 3b). These ARUs had an omnidirectional microphone with a windscreen and were recorded continuously at 48 kHz during the study period. Due to extended battery life and memory capabilities over the Audiomoths, the Solar BARs were only checked once, at the end of the study period. One Kestrel Drop D2 device was set at each ARU to record ambient temperature (in degree Celsius) and relative humidity (in percentage) every 10 min.

### Data processing

#### Acoustic recording units

We segmented each ARU recording into 1‐min segments (1440 samples per device per day) using the *warbleR* package in R (Araya‐Salas & Smith‐Vidaurre, 2017). Samples that were shorter than 1 min were excluded from analyses to ensure consistent calculation of acoustic indices. We then calculated SPL using the *warbleR* package and NDSI using the *soundecology* package (Appendix S1; Villanueva‐Rivera & Pijanowski, 2018). We calculated NDSI as (biophony − anthrophony)/(biophony + anthrophony) (Kasten et al., 2012). We limited the maximum frequency in our analyses to 8 kHz to ensure consistency between devices. Therefore, SPL was the force of all sounds up to 8 kHz, and the biophony for NDSI was maximized at 8 kHz.

#### WCS use

We used the Microsoft MegaDetector artificial intelligence to identify and remove false captures from camera data (Beery et al., 2019). We set confidence thresholds of 55% for animals, 55% for people, 55% for vehicles, and 60% for empty images based on a preliminary study conducted using camera data from the same locations (Scognamillo et al., 2023). These thresholds achieved accuracy levels of 95%–98% compared to hand‐sorted photographs. We labeled all photographs that were identified as empty but not as any other category as false captures and manually sorted the remaining photographs by species and number of individuals using the program Timelapse2 (Greenberg et al., 2019). When an animal in a photo could not be identified, we classified it to the lowest taxonomic level. Once photos were sorted, we identified how each non‐rodent mammal interacted with a WCS, following the protocol of Roy et al. (2024). Using a 30‐min interval to establish independent events, we recorded the duration of interaction and categorized WCS interactions into four ordered categories based on the level of interaction with a WCS: a full crossing where an animal was seen crossing from one side of a WCS to another, an entry/exit where an animal was seen entering a WCS and exiting on the same side, an approach where an animal was seen approaching the WCS but did not enter the WCS, and no interaction where an animal was seen on camera but did not approach the WCS. Duration was calculated in minutes based on the difference between the time of the last photograph and the first photograph of an individual during an interaction event. For this study, we focused our analyses on opossums because they are common in the study area, regularly use wildlife crossings in the region (Roy et al., 2024), and are thought to be disturbance‐tolerant with respect to roads, making them an ideal species to test our framework.

### Statistical analyses

#### Variable selection

Camera trap data, acoustic samples, temperature, and humidity data were recorded at different time intervals, so we used different methods to assign values to samples. When we assessed spatiotemporal variation in SPL and NDSI, we assigned an acoustic sample the nearest temperature and humidity value from the device at the same location. When we assessed how anthrophony impacted opossum WCS use, interaction events typically lasted between a few seconds and a few hours, so we modeled chronic noise using the median of SPL and NDSI and acute noise using the maximum of SPL and minimum of NDSI over the duration of the interaction. For temperature and humidity, we modeled the mean over the duration of the interaction. For a complete description of the selection of covariates, see Appendix S2.

#### Acoustic profile of WCSs

We analyzed the spatiotemporal variation in SPL and NDSI using a randomized block (RBD) arrangement of factors with repeated measures. The blocking effect was WCS, and we computed an hourly average for each device for each day as the unit for repeated measures. We assessed the effects of WCS, position in the WCS (road, WCS entrance, or WCS middle), and time of day (in hours) on variation in SPL and NDSI. All factors were analyzed as fixed effects, and temperature and humidity were included as covariates. All relevant two‐way interactions were included in the model (Kirk, 1995). Non‐significant interactions were removed before assessing the final model. The indices of anthrophony (SPL and NDSI) were the response variables, and both were assessed in an analysis of covariance (ANCOVA) and linear regression framework. SPL was approximately normally distributed, so we analyzed it using the *lm* function in the *stats* package in Program R v4.4.1 (R Core Team, 2024). The NDSI is bounded between −1 and 1, so we transformed it using an (NDSI + 1)/2 transformation (Fairbrass et al., 2017) and analyzed it using a beta error distribution and a logit link using the *betareg* package in Program R (Cribari‐Neto & Zeileis, 2010). The beta distribution effectively models proportions and continuous data with finite limits (Ferrari & Cribari‐Neto, 2004), making beta regression an effective method for modeling NDSI (Fairbrass et al., 2017).

#### Impact of anthrophony on WCS use

For the interaction analysis, we assessed how both the interaction type and duration of the interaction were affected by anthrophony. Opossums made up 67% of the dataset (*n* = 732 out of 1097 detections). For crossing type, we used the proportional odds model in ordinal logistic regression (Ananth & Kleinbaum, 1997; Greenland, 1994). The proportional odds model requires that there is no sparseness in categories of fixed effects (in this case WCS by crossing interaction type) so we combined the entry/exit and approach interactions into a single category, labeled “failed crossings” (Hosmer Jr. et al., 2013, Chap. 8). This gave us three categories: no interaction, failed crossing, and successful crossing. Predictors in this model included both acute (max/min) and chronic (median) versions of SPL and NDSI to represent anthrophony, WCS, temperature, humidity, and duration of event (Table 3). WCS was included because it has been shown that structural differences in WCSs influence crossing rates for mammals in Texas (Yamashita, Scognamillo, et al., 2025).

**TABLE 3:** List of variables used in models on type of interaction and duration of a crossing event, including details about whether they were included as a response or predictor variable, the type of transformation they received, and the mean ± SD of continuous predictors.

Variable name	Variable type		Transformation	Values
	Crossing type	Duration		
Crossing type	Response	…	Logit	No interaction ➔ Failed crossing ➔ successful crossing
Duration	Predictor	Response	Log	2.53 ± 1.73 min
Mean temperature	Predictor	Predictor	None	19.81 ± 3.93°C
Mean humidity	Predictor	Predictor	None	84.68 ± 12.62%
Median SPL^a^	Predictor	Predictor	None	31.70 ± 5.99 dB
Maximum SPL	Predictor	Predictor	None	38.27 ± 6.05 dB
Median NDSI^b^	Predictor	Predictor	None	0.14 ± 0.62 (unitless)
Minimum NDSI	Predictor	Predictor	None	−0.41 ± 0.52 (unitless)

^a^ Sound pressure level.

^b^ Normalized difference soundscape index.

We used the log(*x* + 1) transformation on the duration of an interaction to reduce skewness and improve model fit. We assessed multicollinearity by calculating the correlation among predictors and removed predictors that had correlations above 0.9 (Kutner et al., 2004). No predictors were removed due to multicollinearity effects. To identify the most parsimonious model, we started with a reduced model with only main effects and tested relevant two‐way interactions one at a time to identify significant interactions (Hosmer Jr. et al., 2013, Chap. 3). Once significant interactions were identified, we examined a full model with all significant interactions for significance and model fit, and non‐significant interactions were removed to produce a preliminary final model.

Once a preliminary final model was identified, we tested the model for the proportional odds assumption using the score test in SAS v9.4 (SAS Institute, Cary, NC, USA; Peterson & Harrell Jr., 1990). If the model violated the proportional odds assumption, we calculated a generalized, nonproportional odds model, which assumes different slopes among the class levels for crossing type. We used this model to test the proportional odds assumption of each predictor. We then created a partial proportional odds model where only those predictors that violated the assumption of proportional odds were modeled as a nonproportional odds model. We compared the model fits of the proportional odds model, nonproportional odds model, and partial proportional odds model using Akaike information criterion (AIC) and likelihood ratio tests and chose the best‐fitting model. Using this final model, we calculated odds ratios and assessed the relative effects of each predictor on the probability of an increasingly successful interaction. We ran all models in SAS using the logistic procedure.

For the duration analysis, we used linear regression to assess how anthrophony affected the time an animal spent at a WCS. We transformed duration using the log(*x* + 1) transformation to reduce skewness and improve the assumptions of normality and homogenous variances (Kutner et al., 2004). Predictors for this model included SPL, NDSI, temperature, humidity, and WCS. Multicollinearity among predictors was assessed using the same methods as above, and no predictors were removed as part of this assessment. We tested how the inclusion of appropriate, ecologically relevant predictors impacted the fit of the model and removed those interactions that were non‐significant to identify a final model (Hosmer Jr. et al., 2013). All models were run in SAS using the MIXED procedure. All figures were produced using the *ggplot2* package in Program R (Wickham, 2016).

## RESULTS

### Acoustic profile of WCSs

**FIGURE 4:**
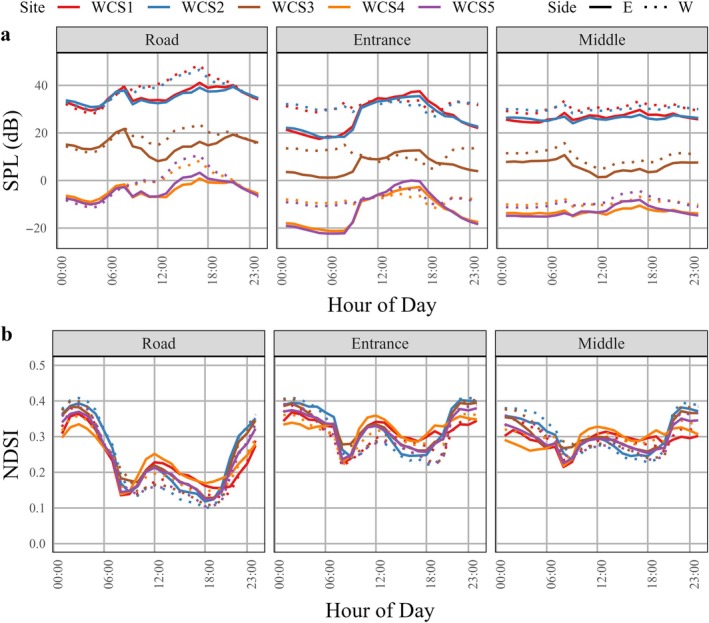
The effect of wildlife crossing structure (WCS), position (road, entrance, or middle of the WCS on the west and east sides of the road), and time‐of‐day (by hour) on (a) sound pressure level (SPL) and (b) normalized difference soundscape index (NDSI) at WCSs on Farm‐to‐Market (FM) 1847 in Cameron County, Texas, USA. Acoustic recording units were deployed between August and October 2023.

**FIGURE 5:**
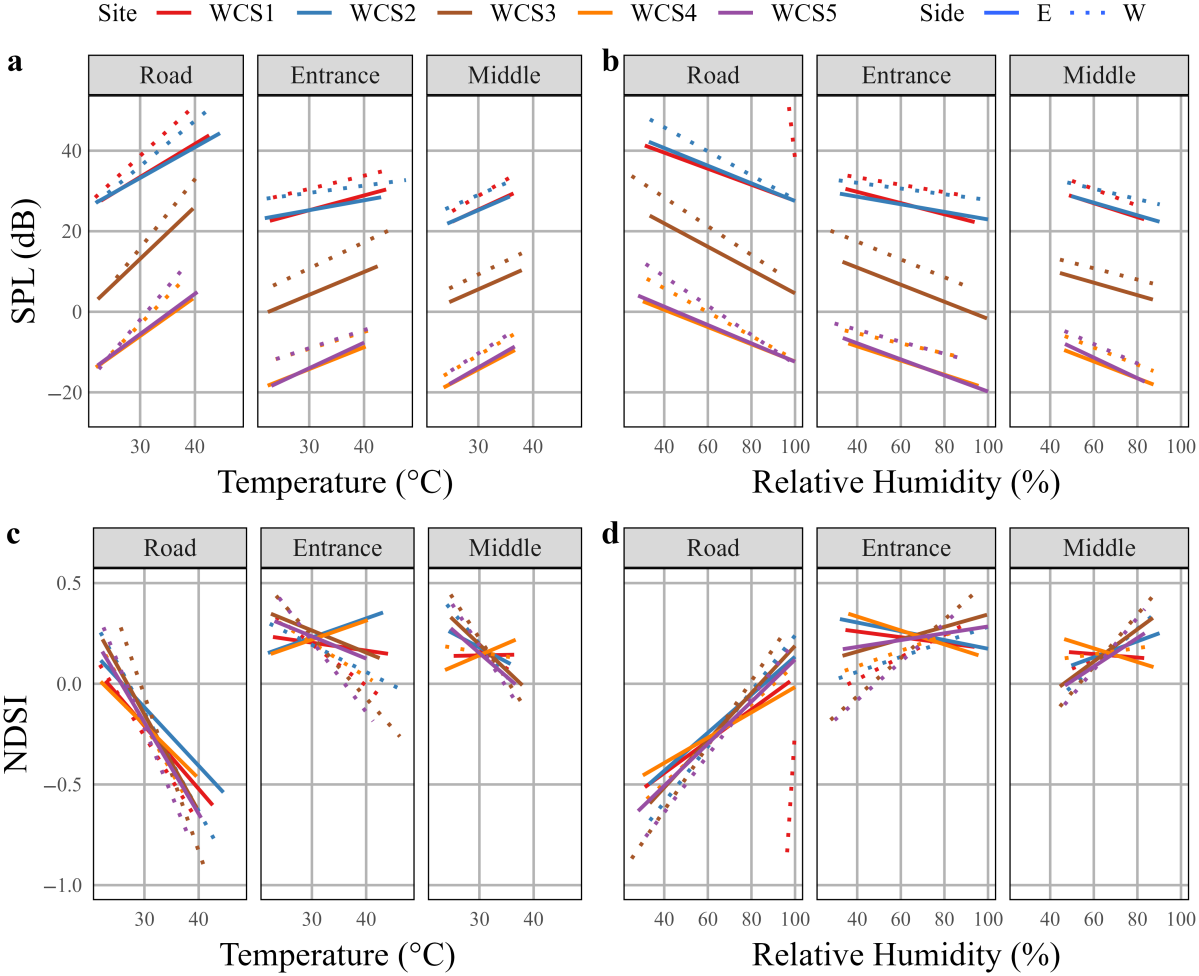
The effect of wildlife crossing structure (WCS), position (road, entrance, or middle of the WCS on the west and east sides of the road), temperature, and humidity on sound pressure level (SPL) and normalized difference soundscape index (NDSI) showing the effect of temperature (a) and humidity (b) on SPL and the effect of temperature (c) and humidity (d) on NDSI at WCSs on Farm‐to‐Market (FM) 1847 in Cameron County, Texas, USA. Acoustic recording units were deployed in August and October 2023.

The final model of SPL included all interactions except that between humidity and hour, while the final model for NDSI included all interactions (Appendix S2). Generally, it was louder (>SPL) with more vehicle noise (<NDSI) at the road positions compared to the entrance or middle positions (Figure 4, Tables 4 and 5). The loudest WCSs were WCS1 and WCS2, with SPL levels on average being between 25 and 40 dB greater than at WCS3, 4, and 5. Generally, vehicle noise levels were greater during the day, although there was much less variability in SPL levels throughout the day at the entrance and middle positions (Figure 4). At WCS4 and 5, the mean SPL was often below the level of human hearing, 20 μPa (Figure 4a). While there were differences in WCSs and positions, SPL increased by 1.78 dB for a 1°C increase in temperature (Figure 5a) and SPL increased by 0.31 dB for a 1% increase in humidity (Figure 5b). NDSI levels were approximately 0.1 lower at road positions compared to entrance or middle positions, but differences in NDSI levels among WCSs were minimal. The direction of effect of temperature depended on WCS, position, and hour (Figure 5c), which resulted in no overall effect of temperature (Table 5). While the effects of humidity varied with WCS, position, and hour, as humidity increased by 1%, the probability of a greater NDSI value decreased by 1.08% (Figure 5d, Appendix S3). There was the least amount of variation in the effect of temperature and humidity on noise at the middle positions, followed by the entrance and road positions (Figure 5, Appendix S3).

**TABLE 4:** Analysis of covariance (ANCOVA) table for the final model of variation in sound pressure level (SPL) based on wildlife crossing structure (WCS; five levels), position around a WCS (six levels), hour (24 levels), temperature (covariate), humidity (covariate), and the two‐way interactions among predictors.

Effect	SS	df	*F* value	*p* value^a^
WCS	4782	4	15.19	**<0.001**
Position	6200	5	15.76	**<0.001**
Hour	19,460	23	10.75	**<0.001**
Temperature	2862	1	36.37	**<0.001**
Humidity	2325	1	29.54	**<0.001**
WCS × hour	11,705	92	1.62	**<0.001**
WCS × temperature	2874	4	9.13	**<0.001**
WCS × humidity	5405	4	17.17	**<0.001**
Position × hour	37,831	115	4.18	**<0.001**
Position × temperature	12,601	5	32.02	**<0.001**
Position × humidity	2268	5	5.76	**<0.001**
Hour × temperature	15,986	23	8.83	**<0.001**
Temperature × humidity	1577	1	20.04	**<0.001**
Residual	1,418,491	18,024		

^a^ Statistically significant effects (at the 0.05 level) are shown in bold.

**TABLE 5:** Analysis of covariance (ANCOVA) table for model of variation in normalized difference soundscape index (NDSI^a^) based on wildlife crossing structure (WCS; five levels), position around a WCS (six levels), hour (24 levels), temperature (covariate), humidity (covariate), and the two‐way interactions among predictors.

Effect	df	χ^b^ value	*p* value^b^
WCS	4	59.16	**<0.001**
Position	5	110.64	**<0.001**
Hour	23	135.67	**<0.001**
Temperature	1	0.02	0.892
Humidity	1	20.74	**<0.001**
WCS × hour	92	1541.51	**<0.001**
WCS × temperature	4	154.58	**<0.001**
WCS × humidity	4	38.42	**<0.001**
Position × hour	115	2349.55	**<0.001**
Position × temperature	5	41.98	**<0.001**
Position × humidity	5	166.39	**<0.001**
Hour × temperature	23	247.43	**<0.001**
Hour × humidity	23	70.58	**<0.001**
Temperature × humidity	1	23.99	**<0.001**

^a^ NDSI is a bounded variable, so logistic regression with a beta error distribution was used to estimate effect sizes.

^b^ Statistically significant effects (at the 0.05 level) are shown in bold.

### Impact of anthrophony on WCS use

We detected 13 mammal species on camera traps during the study period (Table 6) and classified 1097 unique events, 732 of which were opossums. Our final model for crossing success was the partial proportional odds model and included interactions between duration and humidity, duration and minimum NDSI, humidity and median SPL, humidity and maximum SPL, and humidity and minimum NDSI (Table 7, Appendix S2). The final model for assessing the impact of anthrophony on the duration an animal spent at a WCS included interactions between WCS and temperature, WCS and median NDSI, WCS and minimum NDSI, temperature and median NDSI, temperature and minimum NDSI, humidity and median NDSI, and humidity and minimum NDSI (Table 8, Appendix S2).

**TABLE 6:** Species detected on camera traps used to assess the impacts of anthrophony on wildlife crossing structure (WCS) use, scientific name, and number of each type of interaction at WCSs along Farm‐to‐Market 1847 in Cameron County, Texas, USA, between March and April 2024.

Common name	Scientific name	Interaction type				Total
		A^a^	B^b^	C^c^	D^d^	
Virginia opossum	*Didelphis virginiana*	343	7	162	220	732
Collared peccary	*Pecari tajacu*	88	0	12	14	114
Northern raccoon	*Procyon lotor*	61	0	7	7	75
Eastern cottontail	*Sylvilagus floridanus*	13	3	5	33	54
Bobcat	*Lynx rufus*	34	3	3	6	46
Domestic cat	*Felis catus*	4	0	21	21	46
Coyote	*Canis latrans*	0	0	2	8	10
Nine‐banded armadillo	*Dasypus novemcinctus*	2	0	0	4	6
Domestic dog	*Canis familiaris*	0	0	1	5	6
Feral hog	*Sus scrofa*	0	0	1	1	2
Striped skunk	*Mephitis mephitis*	0	0	1	1	2
Long‐tailed weasel	*Neogale frenata*	0	0	0	1	1
White‐tailed deer	*Odocoileus virginianus*	0	0	1	0	1

^a^ An “A” interaction is a full crossing, from one side of the road to the other.

^b^ A “B” interaction is an entry and exit where an animal enters a WCS and exits on the same side.

^c^ A “C” interaction is an approach where an animal approaches a WCS but does not enter.

^d^ A “D” interaction is a non‐interaction where the animal was seen in the area but did not approach the WCS.

**TABLE 7:** Analysis of covariance (ANCOVA) table for the final model used to assess the effect of factors on the time a Virginia opossum (*Didelphis virginiana*) spent at a wildlife crossing structure (WCS), computed using the MIXED procedure in SAS v9.4 (SAS Institute, Cary, NC, USA).

Effect	Num df	Den df	*F* value	*p* value^a^
WCS	4	705	6.3	**<0.001**
Mean temperature	1	705	7.92	**0.005**
Mean humidity	1	705	2.59	0.108
Median SPL^b^	1	705	145.17	**<0.001**
Median NDSI^c^	1	705	410.88	**<0.001**
Median NDSI	1	705	0.75	0.386
Median NDSI	1	705	0.11	0.742
Mean temperature × WCS	4	705	4.48	**0.001**
Median NDSI × WCS	4	705	6.1	**<0.001**
Median NDSI × WCS	4	705	5.22	**<0.001**
Mean temperature × median NDSI	1	705	36.91	**<0.001**
Mean temperature × median NDSI	1	705	16.43	**<0.001**
Mean humidity × median NDSI	1	705	8.62	**0.003**
Mean humidity × median NDSI	1	705	5.67	**0.018**

^a^ Statistically significant effects (at the 0.05 level) are shown in bold.

^b^ Sound pressure level.

^c^ Normalized difference soundscape index.

**TABLE 8:** Full ANOVA tables for the final model used to assess the effect of factors on the probability of a more successful crossing by Virginia opossum (*Didelphis virginiana*) at a wildlife crossing structure (WCS).

Effect	df	Wald χ^a^	*p* value^b^
WCS	8	72.8463	**<0.0001**
Duration^c^	1	0.0087	0.9255
Mean temperature	1	17.1847	**<0.0001**
Mean humidity	1	0	0.9947
Median SPL^a^	1	2.8308	0.0925
Median NDSI^d^	1	2.6476	0.1037
Median NDSI	1	0.0066	0.9351
Median NDSI	1	1.4261	0.2324
Duration × mean humidity	1	1.9624	0.1613
Duration × median NDSI	1	9.1595	**0.0025**
Mean humidity × median SPL	1	3.341	0.0676
Mean humidity × median NDSI	1	2.6694	0.1023
Mean humidity × median NDSI	1	1.0624	0.3027

^a^ Sound pressure level (dB).

^b^ Statistically significant effects (at the 0.05 level) are shown in bold.

^c^ Duration (in minutes) is log‐transformed.

^d^ Normalized difference soundscape index.

**FIGURE 6:**
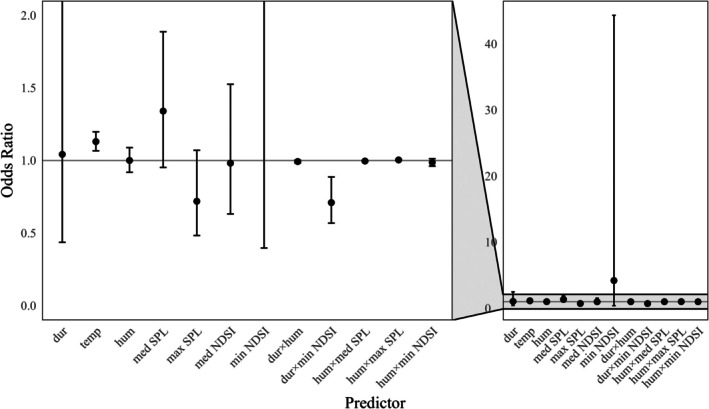
Odds ratios plus 95% CIs for each predictor of the odds of a more successful crossing event by an opossum showing the effects of duration (dur), temperature (temp), humidity (hum), median sound pressure level (med SPL), maximum SPL (max SPL), median normalized difference soundscape index (med NDSI), minimum NDSI (min NDSI), and relevant interactions among predictors at five wildlife crossing structures on Farm‐to‐Market 1847 in Cameron County, Texas, USA between March and April 2024. Odds ratios are percent deviations from 1 and represent the percent change in the odds of a more successful crossing event for a one‐unit increase in the predictor. The left figure is zoomed in from the gray area on the right figure.

The odds of a more successful crossing event were 13% greater with a 1°C increase in temperature (Figure 6, Table 7). The interaction between duration and minimum NDSI represented an interference interaction between predictors, so as animals spent more time at a WCS, the effect of anthrophony on the probability of a more successful crossing event was reduced (Appendix S3). Alternatively, when it was quieter, there was less of a positive effect of duration on the probability of a more successful crossing event.

**FIGURE 7:**
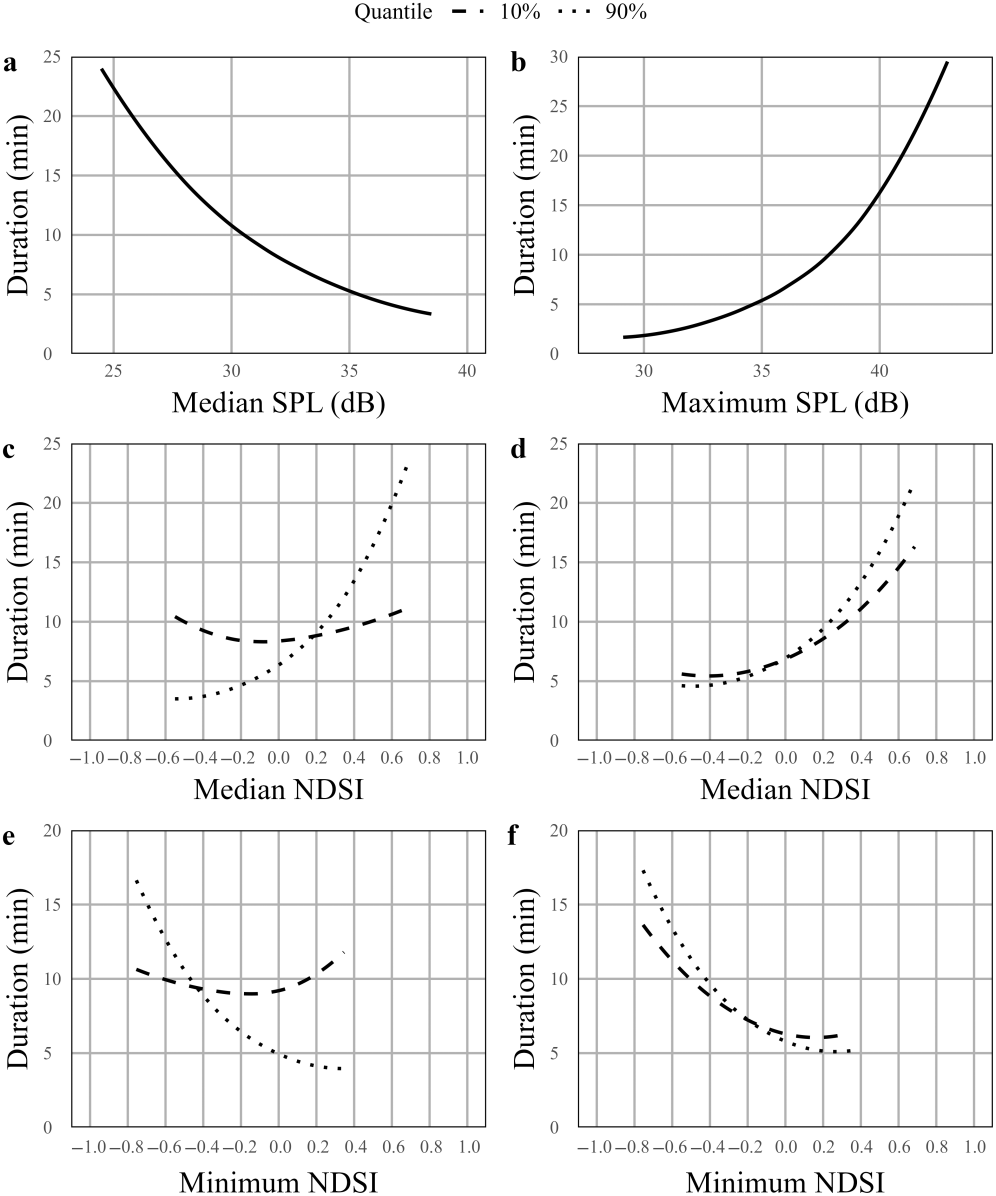
Conditional effects of anthrophony on the time an opossum spent at a wildlife crossing structure (WCS) located on Farm‐to‐Market 1847 between March and April 2024 showing the effect of (a) median sound pressure level (SPL), (b) maximum SPL, (c) median normalized difference soundscape index (NDSI) for varying levels of temperature, (d) median NDSI for varying levels of relative humidity, (e) minimum NDSI for varying levels of temperature, and (f) minimum NDSI for varying levels of relative humidity. To account for interactions between NDSI and temperature/humidity, predicted values were calculated at the 10% (dashed lines) and 90% (dotted lines) quantiles of temperature/humidity instead of the mean of each. Predicted relationships were averaged across WCS.

The time an opossum spent at a WCS decreased by 14.3% for a 1 dB increase in median SPL during that interaction (*p* < 0.001; Table 8, Figure 7a). Maximum SPL resulted in a 25.7% increase in the duration of an interaction for a 1 dB increase in maximum SPL (*p* < 0.001; Figure 7b).

Generally, duration decreased with temperature and humidity, with similar trends observed across the range of median and minimum NDSI (Table 8, Appendix S3). Median NDSI had a positive relationship with duration, with this relationship being stronger when it was warmer and more humid (Figure 7c,d). Minimum NDSI had a mixed relationship with duration that depended on the levels of temperature and humidity. In cool temperatures, duration increased with minimum NDSI, but when it was warmer, duration decreased with minimum NDSI (Figure 7e,f). Duration generally decreased with NDSI across all levels of humidity, but the relationship was stronger when it was more humid (Appendix S3).

## DISCUSSION

In this work, we developed a framework for assessing the impacts of anthrophony on WCS use by first identifying how noise varies in and among WCSs and then assessing how noise impacts the time spent at WCSs and the probability of successful crossings. The entrance and middle of WCSs were approximately 25 dB quieter with NDSI values 0.1 greater than the road surface, while smaller WCSs and those with lower traffic were 15 dB quieter than larger, higher traffic WCSs. NDSI, however, was similar between WCSs, indicating that the source of the noise was likely the same across WCSs. Anthrophony did impact how opossums interacted with WCSs, with opossums spending more time at WCSs when the median noise level was lower, but also when the peak noise was greater. Therefore, smaller WCSs with less traffic may provide better acoustic conditions for opossums to use WCSs.

While we only examined five WCSs in this study, all in South Texas, we identified several patterns in the acoustic conditions of WCSs. First, the WCS entrance and inside the WCS were much quieter compared to the road, indicating that WCSs located below road grade (as all of our WCSs were) may help to isolate WCSs from vehicle noises. Additionally, we found a few differences in the road noise at the entrance compared to the middle of a WCS, indicating that road noise that does get to the WCS also reaches the center of the WCS without a significant drop in amplitude. Second, the orientation of the WCS in relation to the primary wind direction may impact how loud the WCSs were. In our study, the west side of the WCS was generally louder than the east side, a result that is likely due to wind in South Texas often blowing from the Gulf of Mexico (east of the study area; Rand et al., 2020), which may amplify vehicle noise on the west side of the highway. Finally, anthrophony varied across the diel cycle, as expected, with it being up to 25 dB louder during the day than at night. Human activity primarily occurs during the day, so increased anthrophony during the day was an expected result. The contradictory result of lower noise levels when humidity was greater may have been due to humidity being lowest during the daytime, when vehicle traffic is generally greatest. At night, when humidity increases, fewer vehicles produce less noise. Therefore, for vehicle traffic sources of anthrophony, humidity likely plays a reduced role compared to temperature in affecting how anthrophony impacts WCS use.

These results also point to potential influences of WCS placement and design on how anthrophony might affect WCS use. The smallest but most urbanized WCS with the highest traffic, WCS1, was the loudest WCS followed by WCS2, which has similar vehicle traffic as WCS3, WCS4, and WCS5 but was the largest WCS of the sites studied. While vehicle traffic is likely the primary driver of anthrophony in a WCS, larger WCSs may allow more noise to enter the WCS even when the traffic volume is the same. Additionally, while we found large differences in SPL between WCSs, the NDSI levels were similar, indicating that the source of the noise is likely similar for all WCSs, despite the amplitude of the noise being different. Therefore, while larger WCSs are often considered to be the most effective (Gagnon et al., 2011; Grilo et al., 2008) and provide safe passage for the most species (Clevenger & Waltho, 2005), it may be important to consider the target species in determining WCS size. If anthrophony is a concern, building WCSs that are large enough to allow passage while minimizing anthrophony or using noise‐reducing materials in construction may provide the greatest benefit to the target species.

Anthrophony had a significant impact on both the time an animal spent at a WCS and how it interacted with a WCS. High values of SPL and low values of NDSI indicate greater amounts of anthrophony (Fairbrass et al., 2017); our results indicate that opossums spent more time at WCSs where there is, on average, less noise (chronic signal) but also when peak noise is greatest (acute signal). Average noise is more representative of chronic noise conditions, while peak noise can represent acute noise conditions (Masud et al., 2020)—when average noise is greater, opossums are less likely to stay at WCSs; yet, the intermittent noise did increase the duration at WCSs. This increased time spent at WCSs may indicate that opossums are willing to wait for vehicles to pass when quieter conditions would allow for safer passage through the WCS (Francis & Barber, 2013) or that intermittent vehicles represent a disturbance that increases indecision about crossing. Increased time spent at a WCS does mean more opportunity for a quieter period and therefore may lead to an increased probability of a successful crossing. However, if chronic noise conditions are too great, opossums may avoid using WCSs. More studies on animal behavior at WCSs are needed to identify how vehicle presence impacts crossing decisions.

Opossums are known to be well adapted to human‐dominated environments (Fidino et al., 2016; Lombardi et al., 2024; Veon et al., 2023) and may easily habituate to anthropogenic disturbance, including road noise. Therefore, tolerance of anthrophony and waiting for a quieter time to cross roads may be a behavioral response that allows opossums to persist in human‐dominated landscapes and near roads. For species that are less tolerant of anthropogenic disturbance, such as bobcats (*Lynx rufus*), ocelots, mountain lions (*Puma concolor*), or grizzly bears (*Ursus arctos*; Crooks, 2002, Short et al., 2024), road noise may serve as a greater barrier to WCS use. These species generally prefer to use either large overpass‐type WCSs or box culverts, similar to those in this study (Clevenger & Waltho, 2000; Grilo et al., 2008; Huijser et al., 2011), indicating that anthrophony may play an important role in determining WCS effectiveness for those species. As an example, one ocelot was detected in November 2023 at WCS2, but this individual did not successfully cross, potentially because high levels of anthrophony at the time deterred the individual from crossing. Designing WCSs that reduce or mask road noise will likely contribute to greater WCS use by all species, including target species, which are often sensitive to roads and urbanization. Despite the opossum's known tolerance of human disturbance, their preference for crossing at times of reduced anthrophony indicates that anthrophony likely plays a strong role in determining successful crossing events at WCSs.

In this study, we examined five WCSs with similar structural characteristics in a small geographic extent; yet, there were clear differences in anthrophony levels within the different WCSs. The proximity of the WCSs allowed for a direct comparison of anthrophony as the WCSs likely experienced similar temporal variations in vehicle traffic patterns. Only WCS1, located in a more urban area with greater traffic, likely experienced different levels of vehicle traffic. Therefore, identified differences in anthrophony among WCSs were likely due to differences in WCS size and surrounding vegetation rather than differences in traffic patterns.

This has powerful implications for WCS design because anthrophony at a WCS may be a result of not only vehicle traffic but also the size, openness, and vegetation composition immediately around WCSs. WCS design considerations typically focus on the size and shape of the WCS and generally fail to consider anthrophony; yet, we have shown how the crossing success of an urban‐tolerant mammal is negatively affected by anthrophony. Oftentimes, transportation planners focus on creating large WCSs and overpasses because these designs benefit a wide variety of species (Clevenger, 2005; Huijser et al., 2011; Mata et al., 2008); yet, large WCSs may not have as many natural sound barriers and experience more anthrophony, which may offset some of the benefits of their size. Therefore, it is critical to incorporate noise mitigation into WCS designs where its size may create acoustic conditions that deter use. Finally, larger, bridge‐style underpasses and overpasses likely have different acoustic properties than box culvert underpasses, so anthrophony may impact WCS use at these sites differently than at box culverts and warrant further studies.

Our study only examined the impacts of anthrophony on a single species at five WCSs in South Texas; yet, we demonstrated that acoustic conditions at WCSs are dependent on both vehicle traffic and WCS size and that the anthrophony impacts WCS use by opossums. We documented differences in anthrophony among different WCS designs that were likely due to differences in vehicle traffic and WCS size and showed that opossums were more likely to successfully cross through a WCS when it was quieter. Many factors impact WCS use (van der Ree et al., 2015, Chap. 15); however, we observed negative effects of anthrophony on WCS crossing success for a human‐tolerant species, indicating that anthrophony and vehicle traffic likely play an important role in determining WCS effectiveness at fine spatial and temporal scales. Additionally, experimental studies on anthrophony impacts on WCS use are needed to better understand how animals alter their crossing behavior under different anthrophony conditions. Our study showed that anthrophony plays an important role in determining WCS effectiveness, so examining its effects on more species and more WCSs is critical to creating more effective WCSs. While our study did not examine anthrophony in large WCSs or at overpasses, the framework laid out in this study can be applied to more species and more WCSs to develop a more comprehensive understanding of how anthrophony impacts WCS effectiveness. Therefore, incorporating an assessment of anthrophony at a variety of spatial and temporal scales into WCS monitoring will likely improve our understanding of WCS effectiveness and the ability of WCSs to mitigate road impacts on wildlife.

## AUTHOR CONTRIBUTIONS

*Conceptualization*: Thomas J. Yamashita, Jason V. Lombardi, Ashley M. Tanner, and Evan P. Tanner. *Data curation*: Thomas J. Yamashita. *Formal analysis*: Thomas J. Yamashita, Ashley M. Tanner, and Evan P. Tanner. *Funding*: Daniel G. Scognamillo, Michael E. Tewes, and Jason V. Lombardi. *Investigation*: Thomas J. Yamashita. *Methodology*: Thomas J. Yamashita, Ashley M. Tanner, Evan P. Tanner, and Jason V. Lombardi. *Project administration*: Daniel G. Scognamillo, Michael E. Tewes, and Jason V. Lombardi. *Resources*: Daniel G. Scognamillo, Michael E. Tewes, and Jason V. Lombardi. *Software*: Thomas J. Yamashita. *Supervision*: Jason V. Lombardi and Michael E. Tewes. *Validation*: Thomas J. Yamashita. *Visualization*: Thomas J. Yamashita. *Writing—original draft preparation*: Thomas J. Yamashita. *Writing—reviewing and editing*: All authors.

## FUNDING INFORMATION

This work was funded by the Texas Department of Transportation.

## CONFLICT OF INTEREST STATEMENT

The authors declare no conflicts of interest.

## Supporting information


Appendix S1.

Appendix S2.

Appendix S3.

## Data Availability

Code and a sample of the acoustic data are available in Dryad (Yamashita, Lombardi, et al., 2025; Yamashita, Tanner, et al., 2025; https://doi.org/10.5061/dryad.wwpzgmswk). The functions used for processing camera data are available in the cameraTrapping package on Zenodo (Yamashita, 2025; https://doi.org/10.5281/zenodo.17081060). Camera data contain the presence of federally endangered species—these data and full acoustic data are available upon request to Dr. David Hewitt, Director of the Caesar Kleberg Wildlife Research Institute (CKWRI), at david.hewitt@tamuk.edu; please reference CKWRI manuscript number 25‐107 and include the purpose of your request.

## References

[eap70192-bib-0001] Alcocer, I. , H. Lima , L. S. M. Sugai , and D. Llusia . 2022. “Acoustic Indices as Proxies for Biodiversity: A Meta‐Analysis.” Biological Reviews 97: 2209–2236. 10.1111/brv.12890. 35978471 PMC9804652

[eap70192-bib-0002] Ananth, C. V. , and D. G. Kleinbaum . 1997. “Regression Models for Ordinal Responses: A Review of Methods and Applications.” International Journal of Epidemiology 26: 1323–1333. 10.1093/ije/26.6.1323. 9447413

[eap70192-bib-0003] Andrews, K. M. , P. Nanjappa , and S. P. D. Riley . 2015. Roads and Ecological Infrastructure: Concepts and Applications for Small Mammals. Baltimore: Johns Hopkins University Press.

[eap70192-bib-0004] Araya‐Salas, M. , and G. Smith‐Vidaurre . 2017. “warbleR: An r Package to Streamline Analysis of Animal Acoustic Signals.” Methods in Ecology and Evolution 8: 184–191. 10.1111/2041-210X.12624.

[eap70192-bib-0005] Beery, S. , D. Morris , S. Yang , M. Simon , A. Norouzzadeh , and N. Joshi . 2019. “Efficient Pipeline for Automating Species ID in New Camera Trap Projects.” Biodiversity Information Science and Standards 3: e37222. 10.3897/biss.3.37222.

[eap70192-bib-0006] Berkhout, B. W. , A. Budria , D. W. Thieltges , and H. Slabbekoorn . 2023. “Anthropogenic Noise Pollution and Wildlife Diseases.” Trends in Parasitology 39: 181–190. 10.1016/j.pt.2022.12.002. 36658057

[eap70192-bib-0008] Blackburn, A. , A. M. Veals , M. E. Tewes , D. B. Wester , J. H. Young, Jr. , R. W. DeYoung , and H. L. Perotto‐Baldivieso . 2022. “If you Build it, Will they Come? A Comparative Landscape Analysis of Ocelot Roadkill Locations and Crossing Structures.” PLoS One 17: e0267630. 10.1371/journal.pone.0267630. 35503770 PMC9064106

[eap70192-bib-0009] Blickley, J. L. , and G. L. Patricelli . 2010. “Impacts of Anthropogenic Noise on Wildlife: Research Priorities for the Development of Standards and Mitigation.” Journal of International Wildlife Law & Policy 13: 274–292. 10.1080/13880292.2010.524564.

[eap70192-bib-0010] Bohn, D. A. 1988. “Environmental Effects on the Speed of Sound.” Journal of the Audio Engineering Society 36: 223–231.

[eap70192-bib-0011] Chang, B. , and K. Starcher . 2019. “Evaluation of Wind and Solar Energy Investments in Texas.” Renewable Energy 132: 1348–1359. 10.1016/j.renene.2018.09.037.

[eap70192-bib-0012] Clevenger, A. P. 2005. “Conservation Value of Wildlife Crossings: Measures of Performance and Research Directions.” Gaia‐Ecological Perspectives for Science and Society 14: 124–129. 10.14512/gaia.14.2.12.

[eap70192-bib-0013] Clevenger, A. P. , and N. Waltho . 2000. “Factors Influencing the Effectiveness of Wildlife Underpasses in Banff National Park, Alberta, Canada.” Conservation Biology 14: 47–56. 10.1046/j.1523-1739.2000.00099-085.x.

[eap70192-bib-0014] Clevenger, A. P. , and N. Waltho . 2005. “Performance Indices to Identify Attributes of Highway Crossing Structures Facilitating Movement of Large Mammals.” Biological Conservation 121: 453–464. 10.1016/j.biocon.2004.04.025.

[eap70192-bib-0015] Cogan, T. 2018. Monitoring Wildlife Guards and Crossing Structures on a Divided Highway in South Texas. Masters. Brownsville, TX: University of Texas Rio Grande Valley.

[eap70192-bib-0016] Collins, A. C. , T. W. Vickers , and F. M. Shilling . 2022. “Behavioral Responses to Anthropogenic Noise at Highways Vary across Temporal Scales.” Frontiers in Ecology and Evolution 10: 891595. 10.3389/fevo.2022.891595.

[eap70192-bib-0017] Cooper, D. J. , and J. I. Wagner . 2013. “Tropical Storm Driven Hydrologic Regimes Support *Spartina spartinae* Dominated Prairies in Texas.” Wetlands 33: 1019–1024. 10.1007/s13157-013-0459-0.

[eap70192-bib-0018] Cribari‐Neto, F. , and A. Zeileis . 2010. “Beta Regression in R.” Journal of Statistical Software 34: 1–24. 10.18637/jss.v034.i02.

[eap70192-bib-0019] Crooks, K. R. 2002. “Relative Sensitivities of Mammalian Carnivores to Habitat Fragmentation.” Conservation Biology 16: 488–502. 10.1046/j.1523-1739.2002.00386.x.

[eap70192-bib-0020] da Silva, J. N. , A. Banhos , C. S. de Azevedo , P. Diniz , and C. Duca . 2023. “Highway Noise Decreases the Abundance of an Understory Rainforest Bird.” Emu ‐ Austral Ornithology 123: 303–309. 10.1080/01584197.2023.2253837.

[eap70192-bib-0021] Elliott, L. F. , D. D. Diamond , D. True , C. F. Blodgett , D. Pursell , D. German , and A. Treuer‐Kuehn . 2014. Ecological Mapping Systems of Texas: Summary Report. Austin, TX: Texas Parks & Wildlife Department.

[eap70192-bib-0022] Evans, T. , and J. Cooper . 2012. Influence of Wind Direction on Noise Emission and Propagation from Wind Turbines. Fremantle, Australia: Australian Acoustical Society.

[eap70192-bib-0023] Ewing, K. , and C. Best . 2004. “South Texas Tamaulipan Thornscrub Restoration Experiment Measures Growth of Planted Woody Vegetation.” Ecological Restoration 22: 11–17. 10.3368/er.22.1.11.

[eap70192-bib-0024] Fairbrass, A. J. , P. Rennert , C. Williams , H. Titheridge , and K. E. Jones . 2017. “Biases of Acoustic Indices Measuring Biodiversity in Urban Areas.” Ecological Indicators 83: 169–177. 10.1016/j.ecolind.2017.07.064.

[eap70192-bib-0025] Ferrari, S. , and F. Cribari‐Neto . 2004. “Beta Regression for Modelling Rates and Proportions.” Journal of Applied Statistics 31: 799–815. 10.1080/0266476042000214501.

[eap70192-bib-0026] Fidino, M. A. , E. W. Lehrer , and S. B. Magle . 2016. “Habitat Dynamics of the Virginia Opossum in a Highly Urban Landscape.” The American Midland Naturalist 175: 155–167. 10.1674/0003-0031-175.2.155.

[eap70192-bib-0027] Forman, R. T. T. , D. Sperling , J. A. Bissonette , A. P. Clevenger , C. D. Cutshall , V. H. Dale , L. Fahrig , et al. 2003. Road Ecology: Science and Solutions. Washington DC: Island Press.

[eap70192-bib-0028] Francis, C. D. , and J. R. Barber . 2013. “A Framework for Understanding Noise Impacts on Wildlife: An Urgent Conservation Priority.” Frontiers in Ecology and the Environment 11: 305–313. 10.1890/120183.

[eap70192-bib-0029] Fuller, S. , A. C. Axel , D. Tucker , and S. H. Gage . 2015. “Connecting Soundscape to Landscape: Which Acoustic Index Best Describes Landscape Configuration?” Ecological Indicators 58: 207–215. 10.1016/j.ecolind.2015.05.057.

[eap70192-bib-0030] Gagnon, J. W. , N. L. Dodd , K. S. Ogren , and R. E. Schweinsburg . 2011. “Factors Associated with Use of Wildlife Underpasses and Importance of Long‐Term Monitoring.” The Journal of Wildlife Management 75: 1477–1487. 10.1002/jwmg.160.

[eap70192-bib-0031] Greenberg, S. , T. Godin , and J. Whittington . 2019. “Design Patterns for Wildlife‐Related Camera Trap Image Analysis.” Ecology and Evolution 9: 13706–13730. 10.1002/ece3.5767. 31938476 PMC6953665

[eap70192-bib-0032] Greenland, S. 1994. “Alternative Models for Ordinal Logistic Regression.” Statistics in Medicine 13: 1665–1677. 10.1002/sim.4780131607. 7973242

[eap70192-bib-0033] Grilo, C. , J. A. Bissonette , and M. Santos‐Reis . 2008. “Response of Carnivores to Existing Highway Culverts and Underpasses: Implications for Road Planning and Mitigation.” Biodiversity and Conservation 17: 1685–1699. 10.1007/s10531-008-9374-8.

[eap70192-bib-0034] Grilo, C. , F. Z. Ferreira , and E. Revilla . 2015. “No Evidence of a Threshold in Traffic Volume Affecting Road‐Kill Mortality at a Large Spatio‐Temporal Scale.” Environmental Impact Assessment Review 55: 54–58. 10.1016/j.eiar.2015.07.003.

[eap70192-bib-0036] Hardy, H. C. , D. Telfair , and W. H. Pielemeier . 1942. “The Velocity of Sound in Air.” The Journal of the Acoustical Society of America 13: 226–233. 10.1121/1.1916169.

[eap70192-bib-0037] Harris, C. M. 1966. “Absorption of Sound in Air Versus Humidity and Temperature.” The Journal of the Acoustical Society of America 40: 148–159. 10.1121/1.1910031.

[eap70192-bib-0038] Helldin, J. O. 2022. “Are Several Small Wildlife Crossing Structures Better than a Single Large? Arguments from the Perspective of Large Wildlife Conservation.” Nature Conservation 47: 197–213. 10.3897/natureconservation.47.67979.

[eap70192-bib-0039] Hill, A. P. , P. Prince , E. Piña Covarrubias , C. P. Doncaster , J. L. Snaddon , and A. Rogers . 2018. “AudioMoth: Evaluation of a Smart Open Acoustic Device for Monitoring Biodiversity and the Environment.” Methods in Ecology and Evolution 9: 1199–1211. 10.1111/2041-210x.12955.

[eap70192-bib-0040] Hill, A. P. , P. Prince , J. L. Snaddon , C. P. Doncaster , and A. Rogers . 2019. “AudioMoth: A Low‐Cost Acoustic Device for Monitoring Biodiversity and the Environment.” HardwareX 6: e00073. 10.1016/j.ohx.2019.e00073.

[eap70192-bib-0041] Hosmer, D. W., Jr. , S. Lemeshow , and R. X. Sturdivant . 2013. Applied Logistic Regression, 3rd ed. Hoboken, NJ: John Wiley & Sons, Inc.

[eap70192-bib-0042] Huijser, M. P. , T. D. H. Allen , W. Camel‐Means , K. Paul , and P. Basting . 2011. “Use of Wildlife Crossing Structures on US Highway 93 on the Flathead Indian Reservation.” Intermountain Journal of Sciences 17: 76.

[eap70192-bib-0043] Huijser, M. P. , J. W. Duffield , C. Neher , A. P. Clevenger , and T. McGuire . 2022. Cost‐Benefit Analysis of Mitigation Measures along Highways for Large Animal Species: An Update and an Expansion of the 2009 Model. 701‐18‐803 TO 1 Part 3. Carson City, NV: Nevada Department of Transportation.

[eap70192-bib-0045] Kasten, E. P. , S. H. Gage , J. Fox , and W. Joo . 2012. “The Remote Environmental Assessment laboratory's Acoustic Library: An Archive for Studying Soundscape Ecology.” Ecological Informatics 12: 50–67. 10.1016/j.ecoinf.2012.08.001.

[eap70192-bib-0046] Kirk, R. 1995. Experimental Design: Procedures for the Behavioral Sciences. Pacific Grove, CA: Brooks/Cole Publishing Company.

[eap70192-bib-0047] Kok, A. C. M. , B. W. Berkhout , N. V. Carlson , N. P. Evans , N. Khan , D. A. Potvin , A. N. Radford , et al. 2023. “How Chronic Anthropogenic Noise Can Affect Wildlife Communities.” Frontiers in Ecology and Evolution 11: 1130075. 10.3389/fevo.2023.1130075.

[eap70192-bib-0048] Kutner, M. H. , C. J. Nachtsheim , and J. Neter . 2004. Applied Linear Regression Models, 4th ed. New York, NY: McGraw‐Hill/Irwin.

[eap70192-bib-0049] Lombardi, J. V. , A. M. Haines , G. W. Watts, III , L. I. Grassman, Jr. , J. E. Janečka , A. Caso , S. Carvajal , et al. 2022. “Status and Distribution of Jaguarundi in Texas and Northeastern México: Making the Case for Extirpation and Initiation of Recovery in the United States.” Ecology and Evolution 12: e8642. 10.1002/ece3.8642. 35356557 PMC8937848

[eap70192-bib-0050] Lombardi, J. V. , H. L. Perotto‐Baldivieso , and M. E. Tewes . 2020. “Land Cover Trends in South Texas (1987‐2050): Potential Implications for Wild Felids.” Remote Sensing 12: 659. 10.3390/rs12040659.

[eap70192-bib-0051] Lombardi, J. V. , D. G. Scognamillo , and C. E. Comer . 2024. “Raccoons and Opossums Respond Similarly to High and Low Development in the East Texas Pineywoods.” Food Webs 39: e00350. 10.1016/j.fooweb.2024.e00350.

[eap70192-bib-0052] Luera, P. , and C. A. Gabler . 2022. “Combined Effects of Scarification, Phytohormones, Stratification, and Soil Type on the Germination and/or Seedling Performance of Three Tamaulipan Thornscrub Forest Species.” Plants 11: 2687. 10.3390/plants11202687. 36297711 PMC9610753

[eap70192-bib-0053] Martinez, L. A. , J. V. Lombardi , G. Powers , A. D. Anderson , T. Campbell , and R. R. Lopez . 2024. “Assessing Ecological and Socio‐Political Factors in Site Selection for Ocelot Reintroduction in Texas.” Conservation Science and Practice 6: e13113. 10.1111/csp2.13113.

[eap70192-bib-0054] Masud, N. , L. Hayes , D. Crivelli , S. Grigg , and J. Cable . 2020. “Noise Pollution: Acute Noise Exposure Increases Susceptibility to Disease and Chronic Exposure Reduces Host Survival.” Royal Society Open Science 7: 200172. 10.1098/rsos.200172. 33047012 PMC7540788

[eap70192-bib-0055] Mata, C. , I. Hervás , J. Herranz , F. Suárez , and J. E. Malo . 2008. “Are Motorway Wildlife Passages Worth Building? Vertebrate Use of Road‐Crossing Structures on a Spanish Motorway.” Journal of Environmental Management 88: 407–415. 10.1016/j.jenvman.2007.03.014. 17467145

[eap70192-bib-0056] McClure, C. J. W. 2021. “Knowledge Gaps at the Intersection of Road Noise and Biodiversity.” Global Ecology and Conservation 30: e01750. 10.1016/j.gecco.2021.e01750.

[eap70192-bib-0057] Mohsin, F. , M. Arias , C. Albrecht , K. Wahl , A. Fierro‐Cabo , and B. Christoffersen . 2021. “Species‐Specific Responses to Restoration Interventions in a Tamaulipan Thornforest.” Forest Ecology and Management 491: 119154. 10.1016/j.foreco.2021.119154.

[eap70192-bib-0058] Noss, R. F. , W. J. Platt , B. A. Sorrie , A. S. Weakley , D. B. Means , J. Costanza , and R. K. Peet . 2015. “How Global Biodiversity Hotspots May Go Unrecognized: Lessons from the North American Coastal Plain.” Diversity and Distributions 21: 236–244. 10.1111/ddi.12278.

[eap70192-bib-0059] Palecki, M. , I. Durre , J. Lawrimore , and S. Applequist . 2020. “NOAA's U.S. Climate Normals (1991‐2020): Summary of Monthly Normals.”

[eap70192-bib-0060] Peterson, B. , and F. E. Harrell, Jr. 1990. “Partial Proportional Odds Models for Ordinal Response Variables.” Journal of the Royal Statistical Society: Series C (Applied Statistics) 39: 205–217. 10.2307/2347760.

[eap70192-bib-0061] Pijanowski, B. C. , A. Farina , S. H. Gage , S. L. Dumyahn , and B. L. Krause . 2011. “What Is Soundscape Ecology? An Introduction and Overview of an Emerging New Science.” Landscape Ecology 26: 1213–1232. 10.1007/s10980-011-9600-8.

[eap70192-bib-0062] Pijanowski, B. C. , L. J. Villanueva‐Rivera , S. L. Dumyahn , A. Farina , B. L. Krause , B. M. Napoletano , S. H. Gage , and N. Pieretti . 2011. “Soundscape Ecology: The Science of Sound in the Landscape.” Bioscience 61: 203–216. 10.1525/bio.2011.61.3.6.

[eap70192-bib-0063] Prospathopoulos, J. M. , and S. G. Voutsinas . 2005. “Noise Propagation Issues in Wind Energy Applications.” Journal of Solar Energy Engineering 127: 234–241. 10.1115/1.1862257.

[eap70192-bib-0064] R Core Team . 2024. R: A Language and Environment for Statistical Computing. Vienna: R Foundation for Statistical Computing.

[eap70192-bib-0065] Rand, J. T. , L. A. Kramer , C. P. Garrity , B. D. Hoen , J. E. Diffendorfer , H. E. Hunt , and M. Spears . 2020. “A Continuously Updated, Geospatially Rectified Database of Utility‐Scale Wind Turbines in the United States.” Scientific Data 7: 15. 10.1038/s41597-020-0353-6. 31932591 PMC6957502

[eap70192-bib-0066] Roy, A. R. , K. W. Ryer , M. S. Rahman , J. H. Young, Jr. , and R. J. Kline . 2024. “Road Mitigation Structures Designed for Texas Ocelots: Influence of Structural Characteristics and Environmental Factors on Non‐target Wildlife Usage.” PLoS One 19: e0304857. 10.1371/journal.pone.0304857. 39037978 PMC11262682

[eap70192-bib-0067] Şabikoğlu, İ. , and D. Akbaba Şabikoğlu . 2021. “Comparison of Environment Noise Intensity Using Ios and Android Based Sound Measurement Applications and Commercial Sound Measurement Devices and Obtaining the Measurement Uncertainty.” Yüzüncü Yıl Üniversitesi Fen Bilimleri Enstitüsü Dergisi 26: 69–79. 10.53433/yyufbed.894712.

[eap70192-bib-0069] Schomer, P. D. 2000. “Loudness‐Level Weighting for Environmental Noise Assessment.” Acta Acustica united with Acustica 86: 49–61.

[eap70192-bib-0070] Scognamillo, D. G. , T. J. Yamashita , and M. E. Tewes . 2023. Ocelot and Jaguarundi Monitoring Project: Evaluating the Effectiveness of Wildlife Crossings, Cattle Guards, and Fencing on Road Facilities in Cameron County. Kingsville, TX: Caesar Kleberg Wildlife Research Institute, Texas A&M University.

[eap70192-bib-0071] Shannon, G. , M. F. McKenna , L. M. Angeloni , K. R. Crooks , K. M. Fristrup , E. Brown , K. A. Warner , et al. 2016. “A Synthesis of Two Decades of Research Documenting the Effects of Noise on Wildlife.” Biological Reviews 91: 982–1005. 10.1111/brv.12207. 26118691

[eap70192-bib-0072] Shilling, F. , D. Waetjen , T. Longcore , T. W. Vickers , S. McDowell , A. Oke , A. Bass , and C. Stevens . 2022. Improving Light and Soundscapes for Wildlife Use of Highway Crossing Structures. UC‐ITS‐2020‐03. Davis, CA: UC Davis Institute of Transportation Studies.

[eap70192-bib-0073] Shilling, F. M. , A. Collens , T. Longcore , and W. Vickers . 2020. Understanding Behavioral Responses of Wildlife to Traffic to Improve Mitigation Planning. Final Report. Davis, CA: Institute of Transportation Studies, University of California, Davis.

[eap70192-bib-0074] Short, M. L. , C. N. Service , J. P. Suraci , K. A. Artelle , K. A. Field , and C. T. Darimont . 2024. “Ecology of Fear Alters Behavior of Grizzly Bears Exposed to Bear‐Viewing Ecotourism.” Ecology 105: e4317. 10.1002/ecy.4317. 38687245

[eap70192-bib-0075] Teixeira, F. Z. , A. Kindel , S. M. Hartz , S. Mitchell , and L. Fahrig . 2017. “When Road‐Kill Hotspots Do Not Indicate the Best Sites for Road‐Kill Mitigation.” Journal of Applied Ecology 54: 1544–1551. 10.1111/1365-2664.12870.

[eap70192-bib-0076] Texas Department of Transportation . 2022. Texas Department of Transportation Annual Average Daily Traffic Annuals. Austin, TX: Texas Department of Transportation.

[eap70192-bib-0077] Towsey, M. 2018. The Calculation of Acoustic Indices Derived from Long‐Duration Recordings of the Natural Environment. Brisbane, Australia: Queensland University of Technology.

[eap70192-bib-0078] Towsey, M. , J. Wimmer , I. Williamson , and P. Roe . 2014. “The Use of Acoustic Indices to Determine Avian Species Richness in Audio‐Recordings of the Environment.” Ecological Informatics 21: 110–119. 10.1016/j.ecoinf.2013.11.007.

[eap70192-bib-0079] U.S. Fish & Wildlife Service . 2016. Recovery Plan for the Ocelot (Leopardus pardalis). New Mexico, USA: First Revision. U.S. Fish and Wildlife Service, Southwest Region, Albuquerque.

[eap70192-bib-0080] van der Ree, R. , D. J. Smith , and C. Grilo . 2015. Handbook of Road Ecology. Chichester, Sussex, UK: John Wiley & Sons, Ltd.

[eap70192-bib-0081] Veon, J. T. , E. V. Lassiter , E. Johansson , M. Shaw , L. McTigue , A. Massey , R. Gibson , and B. A. DeGregorio . 2023. “Influence of Human Development and Predators on Patterns of Virginia Opossum Occupancy, Abundance, and Activity.” Journal of Zoology 321: 278–288. 10.1111/jzo.13111.

[eap70192-bib-0082] Villanueva‐Rivera, L. J. , and B. C. Pijanowski . 2018. “Soundecology: Soundscape Ecology.”

[eap70192-bib-0083] Villanueva‐Rivera, L. J. , B. C. Pijanowski , J. Doucette , and B. Pekin . 2011. “A Primer of Acoustic Analysis for Landscape Ecologists.” Landscape Ecology 26: 1233–1246. 10.1007/s10980-011-9636-9.

[eap70192-bib-0084] Wickham, H. 2016. ggplot2: Elegant Graphics for Data Analysis. New York, NY: Springer‐Verlag.

[eap70192-bib-0085] Yamashita, T. 2025. “Tomyamashita/cameraTrapping: Initial Release (v1.0.0).” Zenodo 10.5281/zenodo.17081061

[eap70192-bib-0086] Yamashita, T. , A. Tanner , E. Tanner , D. Scognamillo , M. Tewes , J. Young , and J. Lombardi . 2025. “Data and Code from: At the Intersection of Soundscapes and Roads: Quantifying Anthrophony's Influence on Wildlife Crossing Structure Use [Dataset].” Dryad. 10.5061/dryad.wwpzgmswk 41709727

[eap70192-bib-0087] Yamashita, T. J. , J. V. Lombardi , Z. M. Wardle , M. E. Tewes , and J. H. Young, Jr. 2025. “Differences in Mammal Community Response to Highway Construction along a Small Urban‐Rural Gradient.” Wildlife Biology: e01347. 10.1002/wlb3.01347.

[eap70192-bib-0088] Yamashita, T. J. , H. L. Perotto‐Baldivieso , D. B. Wester , K. W. Ryer , R. J. Kline , M. E. Tewes , J. H. Young , and J. V. Lombardi . 2024. “A Multivariate Approach to Assessing Landscape Structure Effects on Wildlife Crossing Structure Use.” Ecological Processes 13: 76. 10.1186/s13717-024-00555-z.

[eap70192-bib-0089] Yamashita, T. J. , D. G. Scognamillo , K. W. Ryer , R. J. Kline , M. E. Tewes , J. H. Young, Jr. , and J. V. Lombardi . 2025. “Predicting Species Assemblages at Wildlife Crossing Structures Using Multivariate Regression of Principal Coordinates.” PLoS One 10: e0335193. 10.1371/journal.pone.0335193. PMC12551880 41134870

[eap70192-bib-0090] Yamashita, T. J. , D. B. Wester , M. E. Tewes , J. H. Young , and J. V. Lombardi . 2023. “Distinguishing Buildings from Vegetation in an Urban‐Chaparral Mosaic Landscape with LiDAR‐Informed Discriminant Analysis.” Remote Sensing 15: 1703. 10.3390/rs15061703.

