# Supplementary material for: At the intersection of soundscapes and roads: Quantifying anthrophony's influence on wildlife crossing structure use
Source: Ecol Appl. 2026 Feb 19;36(1):e70192. doi: 10.1002/eap.70192 (PMC12917473; doi:10.1002/eap.70192)
Supplement: Supplementary file 1 — Appendix S1. [file EAP-36-e70192-s002.pdf]

**Supporting Information**

At the intersection of soundscapes and roads: Quantifying anthrophony's influence on wildlife crossing structure use

Thomas J. Yamashita, Ashley M. Tanner, Evan P. Tanner, Daniel G. Scognamillo, Michael E. Tewes, John H. Young Jr., and Jason V. Lombardi

*Ecological Applications*

**Appendix S1. An annotated spectrogram with associated sound pressure level and NDSI graphs.**

An example of a spectrogram for a 10-minute segment of a recording from an Audiomoth device set up at Wildlife Crossing Structure (WCS) 1 on Farm-to-Market 1847 in Cameron County, Texas in Fall 2023. Spectrograms were created using the *spectro* function from the *seewave* package (Sueur et al. 2008) and plots were produced using the *ggplot2* package in Program R (Wickham 2016).

References

Sueur, J., T. Aubin, and C. Simonis. 2008. Seewave: A free modular tool for sound analysis and synthesis. Bioacoustics 18(2):213–226. https://doi.org/10.1080/09524622.2008.9753600

Wickham, H., 2016. ggplot2: Elegant graphics for data analysis. Springer-Verlag, New York, New York, USA.

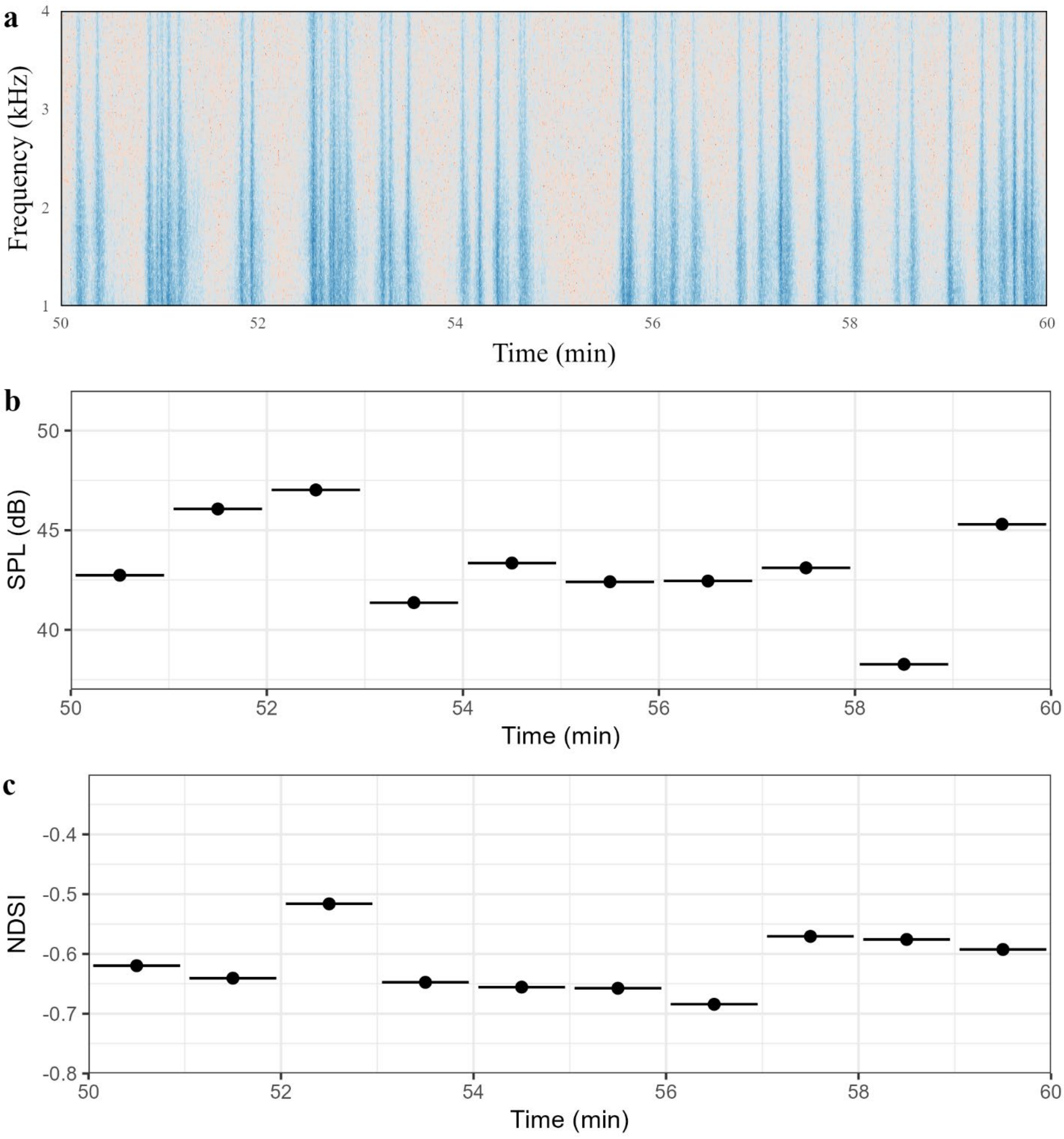


Figure S1. A spectrogram over 10 minutes showing the associated sound pressure level (SPL) and normalized difference soundscape index (NDSI) for one-minute segments over the same span. Blue lines on the spectrogram represent vehicles passing by the Audiomoth.
