# Supplementary material for: At the intersection of soundscapes and roads: Quantifying anthrophony's influence on wildlife crossing structure use
Source: Ecol Appl. 2026 Feb 19;36(1):e70192. doi: 10.1002/eap.70192 (PMC12917473; doi:10.1002/eap.70192)
Supplement: Supplementary file 2 — Appendix S2. [file EAP-36-e70192-s003.pdf]

Supporting Information

At the intersection of soundscapes and roads: Quantifying anthrophony's influence on wildlife crossing structure use

Thomas J. Yamashita, Ashley M. Tanner, Evan P. Tanner, Daniel G. Scognamillo, Michael E. Tewes, John H. Young Jr., and Jason V. Lombardi

Ecological Applications

Appendix S2. Variable selection and modeling results for all analyses.

The following appendix provides the statistical details of the analyses in this manuscript and the necessary tables to interpret F and P values used to assess the significance of predictors used to model differences in sound pressure level (SPL) and normalized difference soundscape index (NDSI) at different positions around wildlife crossing structures (WCS) on Farm-to-Market Road 1847 in Cameron County, Texas in Fall 2023 and the relative effects of SPL and NDSI on the time a Virginia opossum (*Didelphis virginiana*) spent at a WCS and whether it successfully crossed in Spring 2024.

Methods

Variable Selection

Camera trap data, acoustic samples, temperature, and humidity data were recorded at different time intervals, so we used different methods to assign values to samples. When we assessed spatiotemporal variation in SPL and NDSI, we assigned an acoustic sample the nearest temperature and humidity value from the device at the same location. When we assessed how anthrophony impacted opossum WCS use, interaction events typically lasted between a few seconds and a few hours, so we identified the range of acoustic samples, temperature, and

humidity records over the duration of the interaction event and then computed the mean and the 0.0 (minimum), 0.5 (median), and 1.0 (maximum) quantiles for each predictor. We were interested in chronic and acute noise impacts on WCS use, so we selected one measure of the average and one measure of the peak of anthrophony over the duration of the interaction. We examined the distributions of each sample and determined that the median for SPL and NDSI appropriately modeled chronic noise, the maximum of SPL, and the minimum of NDSI represented peak anthrophony (acute noise). There were minimal differences in temperature and humidity among the mean, median, min, and max, so we only modeled the mean of each over the duration of an interaction.

Acoustic profile of wildlife crossing structures

We analyzed the spatiotemporal variation in SPL and NDSI using a randomized block (RBD) arrangement of factors with repeated measures. The blocking effect was WCS, and hour was the unit used for repeated measures. Models containing minute as the repeated measure (the appropriate unit for these data) could not be run due to memory allocation errors in both Program R v4.4.1 and SAS v9.4, so we computed an hourly average for each device for each day before conducting statistical analyses. We assessed the effects of WCS, position in the WCS (road, WCS entrance, or WCS middle), and time of day (hour) on variation in SPL and NDSI. All factors were analyzed as fixed effects and temperature and humidity were included as covariates. All two-way interactions were included in the model (site and position were the only interaction excluded due to RBD design limitations; Kirk 1995). Non-significant interactions were removed before assessing the final model. The indices of anthrophony (SPL and NDSI) were the response variables, and both were assessed in an analysis of covariance (ANCOVA) and linear regression framework. Sound pressure level was approximately normally-distributed so we analyzed it

using the *lm* function in the *stats* package in Program R v4.4.1 (R Core Team 2024). The NDSI is bounded between -1 and 1 so we transformed it using an (NDSI + 1)/2 transformation (Fairbrass et al. 2017) and analyzed it using a beta error distribution and a logit link using the *betareg* package in Program R (Cribari-Neto and Zeileis 2010). The beta distribution effectively models proportions and continuous data with finite limits (Ferrari and Cribari-Neto 2004) making beta regression an effective method for modeling NDSI (Fairbrass et al. 2017).

Results

Acoustic profile of wildlife crossing structures

The interaction between humidity and hour was removed from the final model of SPL due to non-significance (Table S1). There were significant interactions between WCS and hour ($F_{92,18024} = 1.62$, $p < 0.001$) and between position and hour ($F_{115,18024} = 4.18$, $p < 0.001$; Figure 4a) impacting the spatiotemporal variation in SPL. The effect temperature on SPL depended on WCS ($F_{4,18024} = 9.13$, $p < 0.001$), position ($F_{5,18024} = 32.02$, $p < 0.001$), hour ($F_{23,18024} = 8.83$, $p < 0.001$), and humidity ($F_{1,18024} = 20.04$, $p < 0.001$; Main Text Figure 5a). The effect of humidity depended on WCS ($F_{4,18024} = 17.17$, $p < 0.001$), position ($F_{5,18024} = 5.76$, $p < 0.001$), and temperature ($F_{1,18024} = 20.04$, $p < 0.001$; Main Text Figure 5b).

All ecologically relevant interactions were included in the best model for NDSI (Main Text Table 5). There were significant interactions between WCS and hour ($\chi^2_{92,18000} = 1,541,51$, $p < 0.001$) and between position and hour ($\chi^2_{115,18000} = 2,349.55$, $p < 0.001$; Main Text Figure 4b). The effect of temperature depended on WCS ($\chi^2_{4,18000} = 154.58$, $p < 0.001$), position ($\chi^2_{5,18000} = 41.98$, $p < 0.001$), hour ($\chi^2_{23,18000} = 247.43$, $p < 0.001$), and humidity ($\chi^2_{1,18000} = 23.99$, $p < 0.001$; Main Text Figure 5c). The effect of humidity depended on WCS ($\chi^2_{4,18000} = 38.42$, $p < 0.001$),

position ($\chi^2_{5,18000}$ = 166.39, p < 0.001), hour ($\chi^2_{23,18000}$ = 70.58, p < 0.001), and temperature ($\chi^2_{1,18000}$ = 23.99, p < 0.001; Main Text Figure 5d).

Impact of anthrophony on wildlife crossing structure use

The proportional odds model violated the assumption of proportional odds (χ^2_{16} = 78.43, p < 0.001) but after testing individual predictors, only WCS violated this assumption (Wald χ^2_1 = 11.85-31.32, all p < 0.001; Table S4). Therefore, our partial proportional odds model only modeled WCS as nonproportional odds. The partial proportional odds model had the lowest AIC (Appendix S2), and the fit was better than the proportional odds model (χ^2_4 = 55.44, p < 0.001) but not different than the nonproportional odds model (χ^2_{12} = 16.7432, p = 0.160). Therefore, our final model was the partial proportional odds model (Table S4).

References

Cribari-Neto, F., and A. Zeileis. 2010. Beta Regression in R. Journal of Statistical Software **34**:1 - 24. https://doi.org/10.18637/jss.v034.i02.

Fairbrass, A. J., P. Rennert, C. Williams, H. Titheridge, and K. E. Jones. 2017. Biases of acoustic indices measuring biodiversity in urban areas. Ecological Indicators **83**:169-177. https://doi.org/10.1016/j.ecolind.2017.07.064.

Ferrari, S., and F. Cribari-Neto. 2004. Beta Regression for Modelling Rates and Proportions. Journal of Applied Statistics **31**:799-815. https://doi.org/10.1080/0266476042000214501.

Kirk, R., 1995. Experimental Design: Procedures for the Behavioral Sciences. Brooks/Cole Publishing Company, Pacific Grove, CA, USA.

R Core Team. 2024. R: a Language and environment for statistical computing. R Foundation for Statistical Computing, Vienna, Austria.

Table S1. ANCOVA table for the full model of variation in sound pressure level (SPL) based on wildlife crossing structure (WCS; five levels), position around a WCS (six levels), hour (24 levels), temperature (covariate), humidity (covariate), and the two-way interactions among predictors.

Effect	SS	DF	F value	P value[1]
WCS	4455	4	14.14	**< 0.001**
Position	6098	5	15.49	**< 0.001**
Hour	12778	23	7.05	**< 0.001**
Temperature	2126	1	27.00	**< 0.001**
Humidity	678	1	8.61	**0.003**
WCS × Hour	11709	92	1.62	**< 0.001**
WCS × Temperature	3031	4	9.62	**< 0.001**
WCS × Humidity	5093	4	16.17	**< 0.001**
Position × Hour	36137	115	3.99	**< 0.001**
Position × Temperature	12630	5	32.08	**< 0.001**
Position × Humidity	2216	5	5.63	**< 0.001**
Hour × Temperature	13627	23	7.52	**< 0.001**
Hour × Humidity	923	23	0.51	0.975
Temperature × Humidity	699	1	8.87	**0.003**
Residual	1417569	18001		

[1]Statistically significant effects (at the 0.05 level) are shown in bold.

Table S2. ANCOVA table for the full interactions model of time that a Virginia opossum (*Didelphis virginiana*) spent at a wildlife crossing structure (WCS), used to narrow down the list of interactions in the final model computed using the MIXED procedure in SAS v9.4 (SAS Institute, Cary, NC, USA).

Interaction[1]	Num. DF	Den. DF	F value	P value[2]
Mean Temperature × WCS	4	688	4.55	**0.001**
Mean Humidity × WCS	4	688	0.38	0.822
Median SPL[3] × WCS	4	688	0.26	0.902
Median NDSI[4] × WCS	4	688	0.98	0.420
Median NDSI × WCS	4	688	4.56	**0.001**
Median NDSI × WCS	4	688	4.57	**0.001**
Mean Temperature × Mean Humidity	1	688	0.31	0.575
Mean Temperature × Median SPL	1	688	2.43	0.120
Mean Temperature × Median NDSI	1	688	3.25	0.072
Mean Temperature × Median NDSI	1	688	27.59	**< 0.001**
Mean Temperature × Median NDSI	1	688	6.09	**0.014**
Mean Humidity × Median SPL	1	688	3.53	0.061
Mean Humidity × Median NDSI	1	688	0.01	0.941
Mean Humidity × Median NDSI	1	688	4.32	**0.038**
Mean Humidity × Median NDSI	1	688	4.09	**0.044**

[1]Only interaction effects are shown.

[2]Statistically significant effects (at the 0.05 level) are shown in bold.

[3]Sound pressure level.

[4]Normalized difference soundscape index.

Table S3. ANCOVA table for the full interactions model of crossing success probability at a wildlife crossing structure (WCS) by a Virginia opossum (*Didelphis virginiana*), used to narrow down the set of interactions for the preliminary final proportional odds model computed with the LOGISTIC procedure in SAS v9.4 (SAS Institute, Cary, NC, USA).

Interaction[1]	DF	Wald χ^2	P value[4]
Duration[1] × WCS	4	2.286	0.683
Mean Temperature × WCS	4	3.253	0.516
Mean Humidity × WCS	4	3.570	0.467
Median SPL[2] × WCS	4	1.190	0.880
Median NDSI[3] × WCS	4	3.792	0.435
Median NDSI × WCS	4	0.906	0.924
Median NDSI × WCS	4	2.138	0.710
Duration × Mean Temperature	1	0.599	0.439
Duration × Mean Humidity	1	4.243	**0.039**
Duration × Median SPL	1	1.006	0.316
Duration × Median NDSI	1	1.444	0.230
Duration × Median NDSI	1	0.248	0.618
Duration × Median NDSI	1	6.054	**0.014**
Mean Temperature × Mean Humidity	1	0.752	0.386
Mean Temperature × Median SPL	1	0.803	0.370
Mean Temperature × Median NDSI	1	0.248	0.618
Mean Temperature × Median NDSI	1	0.656	0.418
Mean Temperature × Median NDSI	1	0.338	0.561
Mean Humidity × Median SPL	1	5.470	**0.019**
Mean Humidity × Median NDSI	1	4.732	**0.030**
Mean Humidity × Median NDSI	1	1.926	0.165
Mean Humidity × Median NDSI	1	4.732	**0.030**

[1]Duration (min) is log-transformed.

[2] Sound pressure level (dB).

[3]Normalized difference soundscape index.

[4]Statistically significant effects (at the 0.05 level) are shown in bold.

Table S4. Likelihood ratio tests and AIC for the test of the assumption of proportional odds in the model of the effect of factors on Virginia opossum (*Didelphis virginiana*) probability of a successful crossing a wildlife crossing structure (WCS).

Model	AIC	Likelihood ratio test	
		PO	NPO
Proportional odds model (PO)	1416.06	----	----
Nonproportional odds model (NPO)	1375.88	$\chi^2 = 72.1835$<br>$P < 0.001$	----
Partial proportional odds model (PPO)	1368.62	$\chi^2 = 55.4404$<br>$P < 0.001$	$\chi^2 = 16.7432$<br>$P = 0.160$
