# Supplementary material for: At the intersection of soundscapes and roads: Quantifying anthrophony's influence on wildlife crossing structure use
Source: Ecol Appl. 2026 Feb 19;36(1):e70192. doi: 10.1002/eap.70192 (PMC12917473; doi:10.1002/eap.70192)
Supplement: Supplementary file 3 — Appendix S3. [file EAP-36-e70192-s001.pdf]

Supporting Information

At the intersection of soundscapes and roads: Quantifying anthrophony's influence on wildlife crossing structure use

Thomas J. Yamashita, Ashley M. Tanner, Evan P. Tanner, Daniel G. Scognamillo, Michael E. Tewes, John H. Young Jr., and Jason V. Lombardi

Ecological Applications

Appendix S3. Supporting figures for interpretation of statistical interactions.

These figures provide additional information to aid in interpretation of the impacts of temperature and humidity on sound pressure level and normalized difference soundscape index at wildlife crossing structures on Farm-to-Market (FM) Road 1847 in Cameron County, Texas in Fall 2023. Additional figures are provided to highlight differences in the probability of a successful crossing event by a Virginia opossum (*Didelphis virginiana*) at a wildlife crossing structure on FM 1847 in Spring 2024.

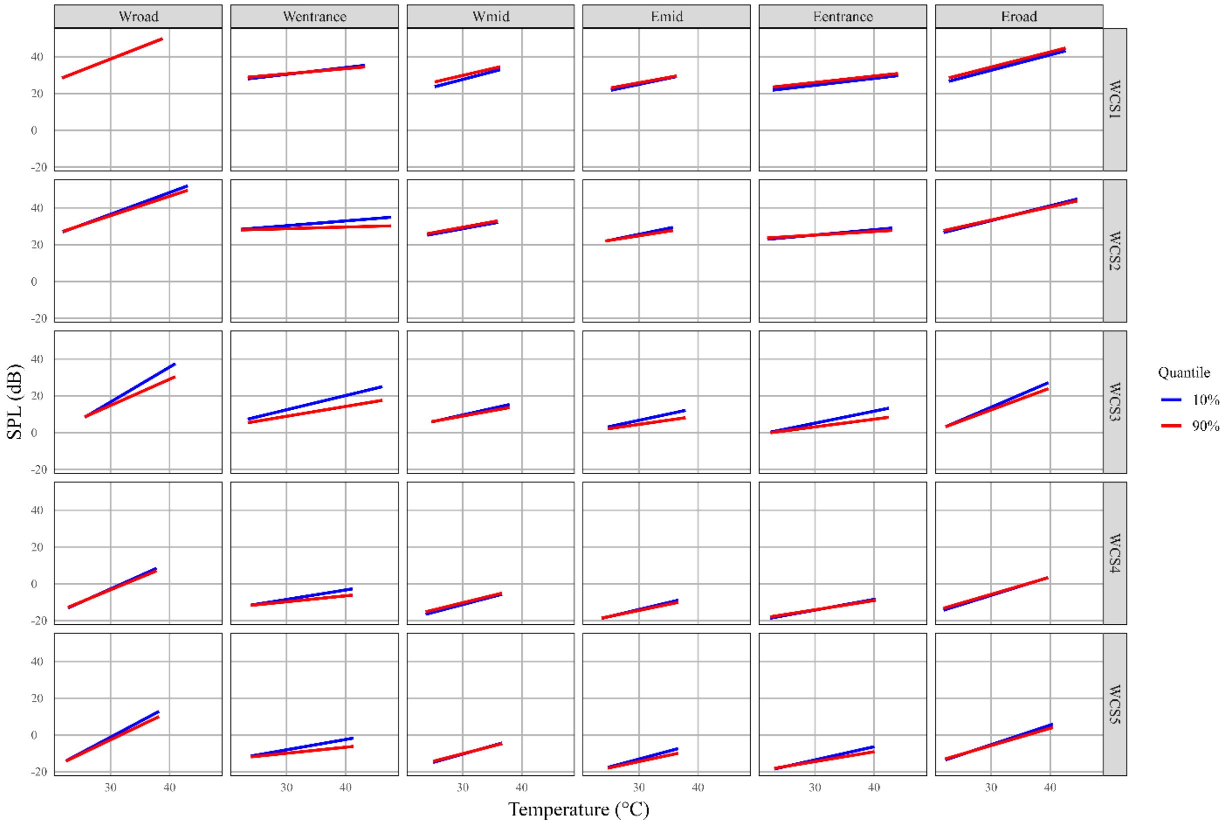

Figure S1: The effect of temperature on sound pressure level (SPL) at the 0.1 and 0.9 quantile of humidity at a given site and position.

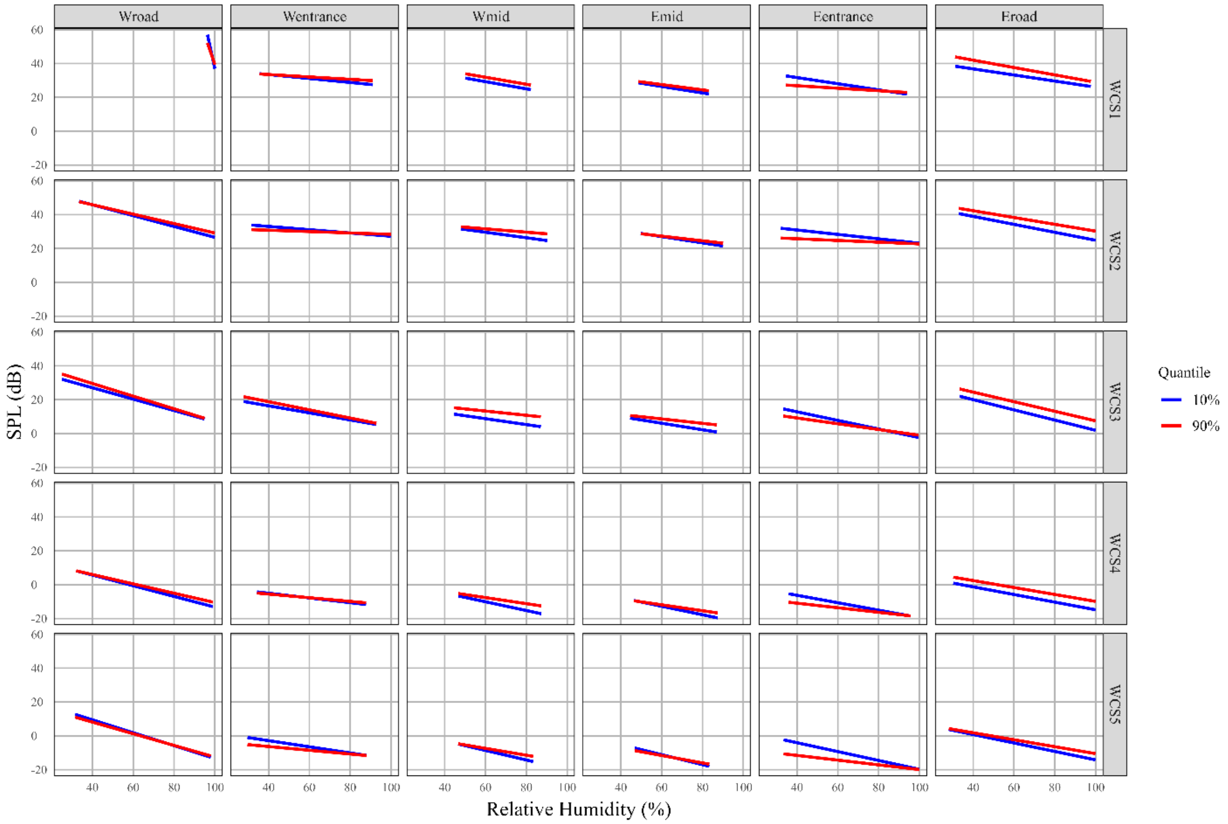

Figure S2: The effect of humidity on sound pressure level (SPL) at the 0.1 and 0.9 quantile of temperature at a given wildlife crossing structure (WCS) and position (east or west side of Farm-to-Market road 1847 and position of the device around a WCS [road, WCS entrance, or middle (mid)]).

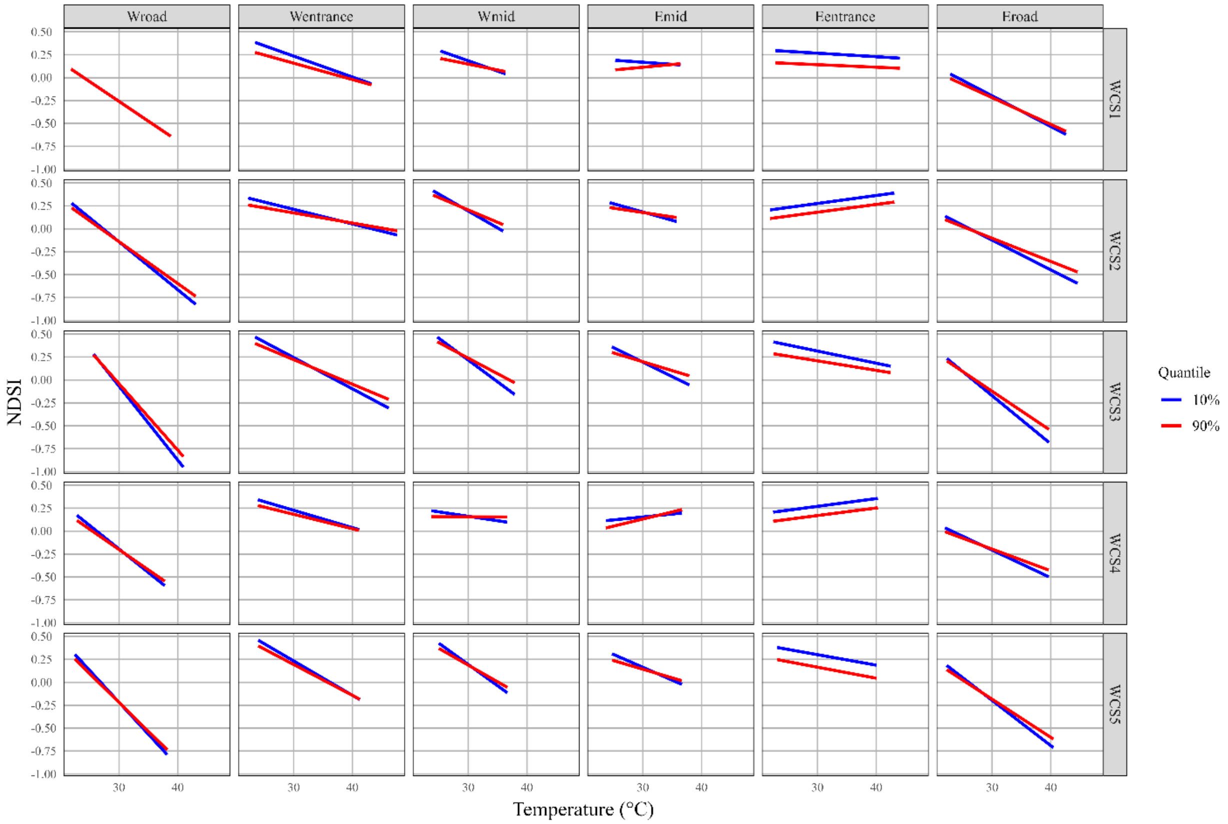

Figure S3: The effect of temperature on normalized difference soundscape index (NDSI) at the 0.1 and 0.9 quantile of humidity at a given wildlife crossing structure (WCS) and position (east or west side of Farm-to-Market road 1847 and position of the device around a WCS [road, WCS entrance, or middle (mid)]).

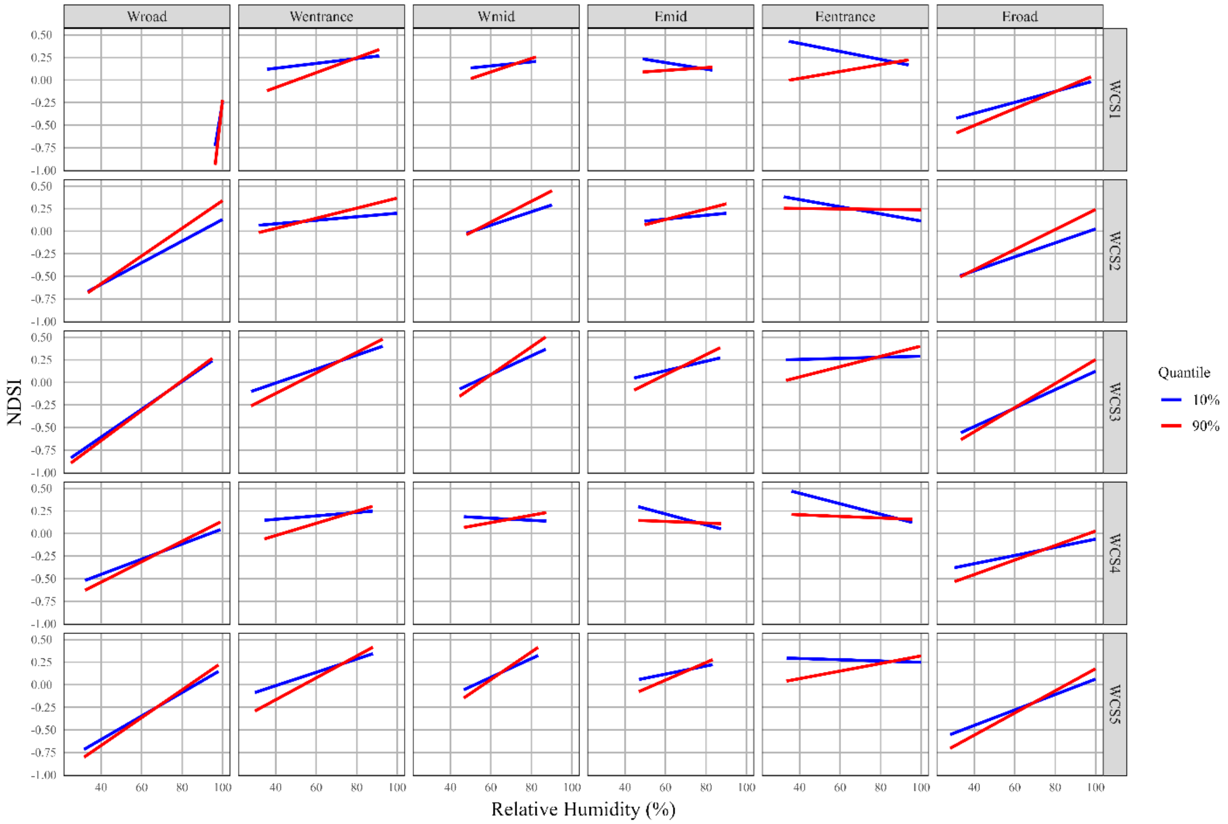

Figure S4: The effect of humidity on normalized difference soundscape index (NDSI) at the 0.1 and 0.9 quantile of temperature at a given wildlife crossing structure (WCS) and position (east or west side of Farm-to-Market road 1847 and position of the device around a WCS [road, WCS entrance, or middle (mid)]).

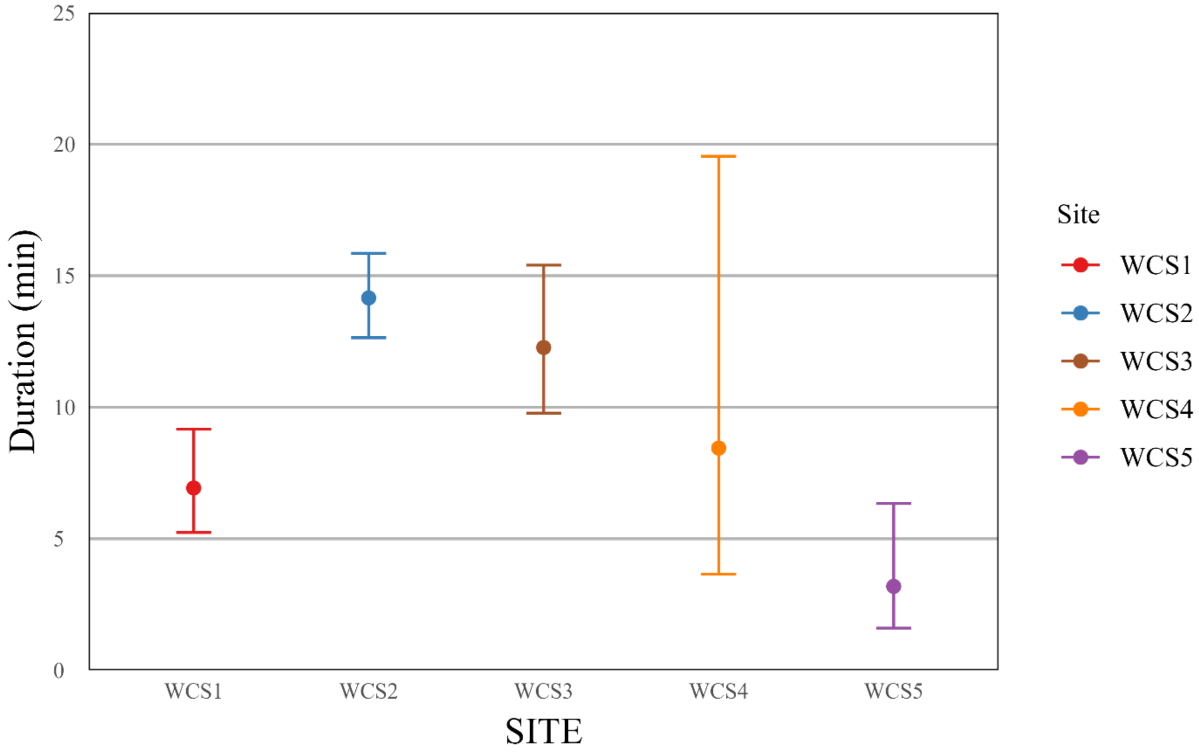

Figure S5. The effect of wildlife crossing structure (WCS) averaged across all other factors on the time a Virginia opossum (*Didelphis virginiana*) spent at a WCS.

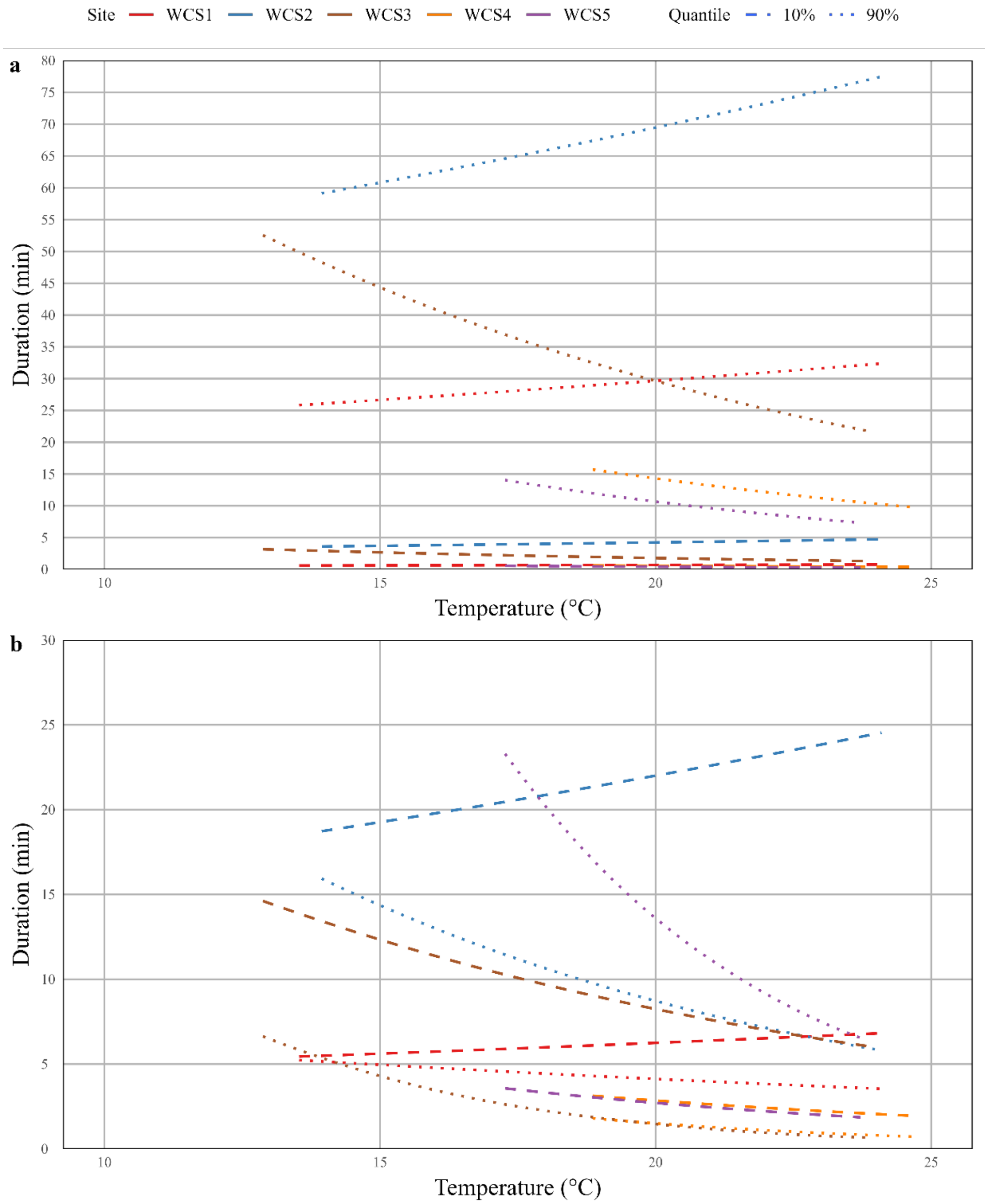

Figure S6. The effect of temperature on the time a Virginia opossum (*Didelphis virginiana*) spent at a wildlife crossing structure (WCS) at different levels of WCS and the 0.1 and 0.9 quantiles of (a) median normalized difference soundscape index (NDSI) and (b) minimum NDSI.

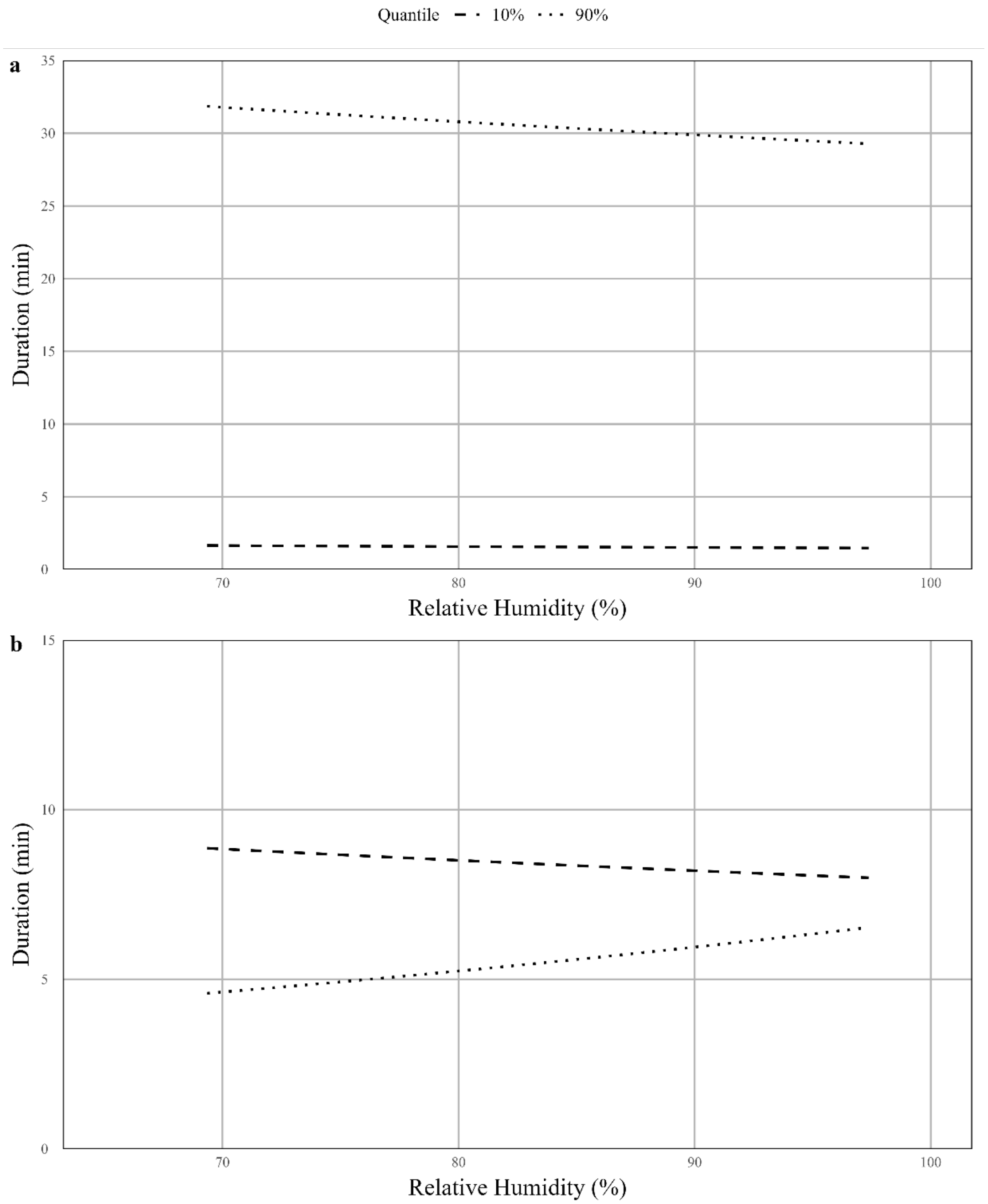

Figure S7. The effect of humidity on the time a Virginia opossum (*Didelphis virginiana*) spent at a wildlife crossing structure (WCS) at the 0.1 and 0.9 quantiles of (a) median normalized difference soundscape index (NDSI) and (b) minimum NDSI.

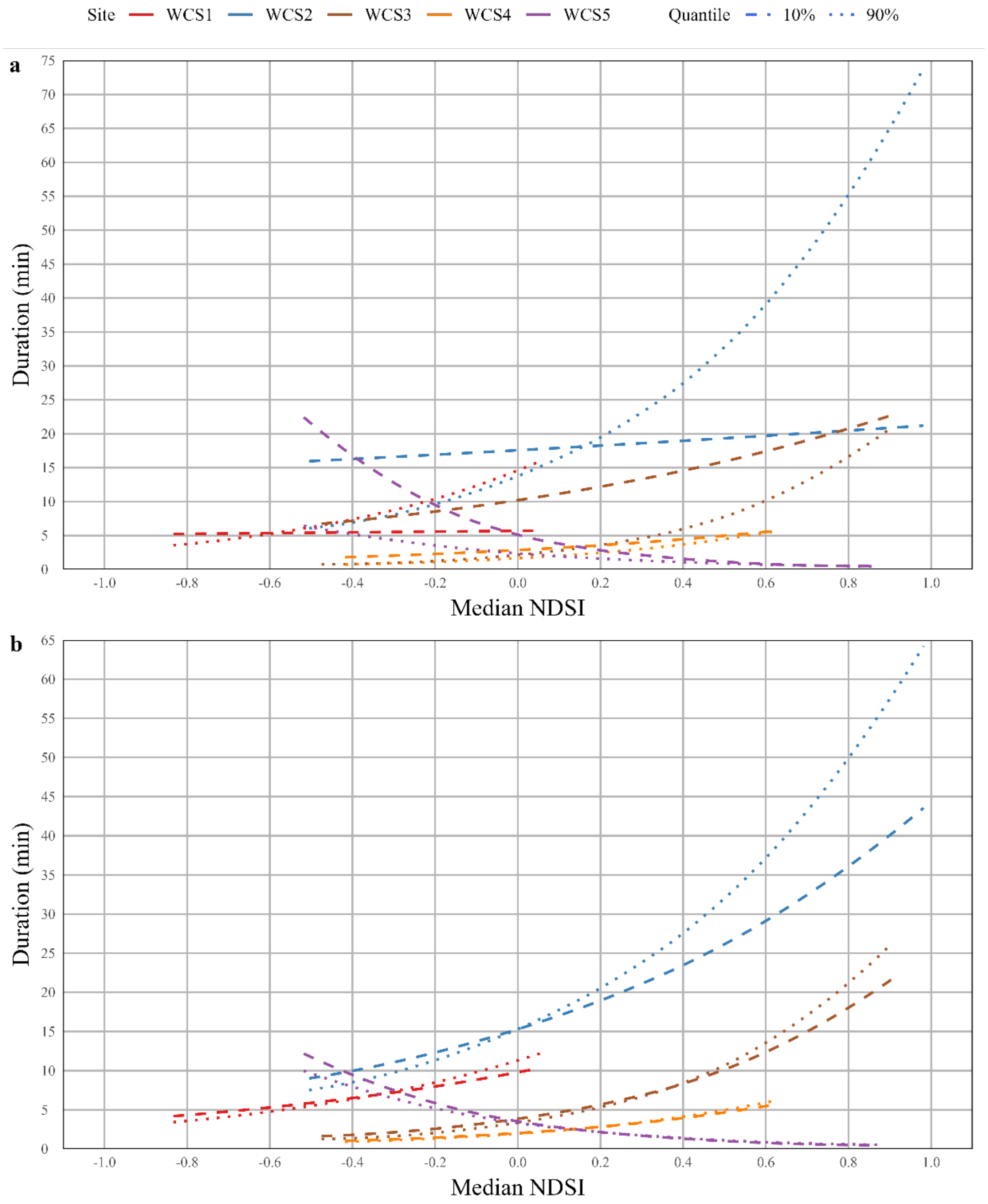

Figure S8. The effect of median normalized difference soundscape index (NDSI) on the time a Virginia opossum (*Didelphis virginiana*) spent at a wildlife crossing structure (WCS) at (a) different levels of WCS and at the 0.1 and 0.9 quantiles of (b) temperature and (c) humidity.

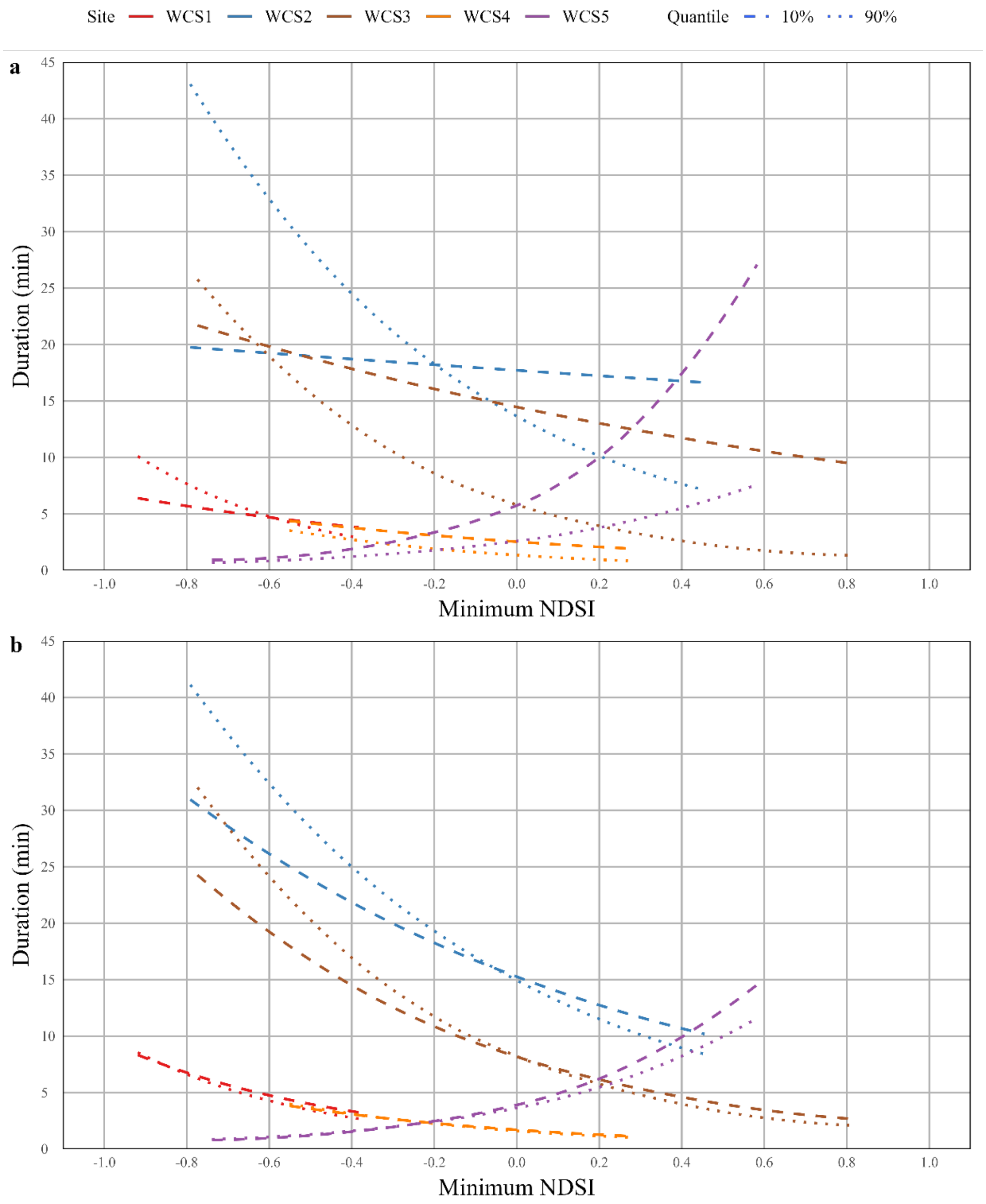

Figure S9. The effect of minimum normalized difference soundscape index (NDSI) on the time a Virginia opossum (*Didelphis virginiana*) spent at a wildlife crossing structure (WCS) at different levels of WCS and at the 0.1 and 0.9 quantiles of (a) temperature and (b) humidity.
